# The Effects of Digital Health Interventions for Family Members in Intensive Care Units: Systematic Review and Meta-Analysis of Randomized Controlled Trials

**DOI:** 10.2196/83294

**Published:** 2026-02-25

**Authors:** Chuchu Zhang, Weijing Sui, Weilin Jiang, Yibing Shen, Zhenzhen Huang, Ran Yan, Jia Yi, Jiayu Zhang, Chaoyi Zhang, Yiyu Zhuang, Xiaoyan Gong

**Affiliations:** 1 Department Nursing Zhejiang University School of Medicine Sir Run Run Shaw Hospital Hangzhou, Zhejiang China

**Keywords:** digital health interventions, intensive care units, family members, psychology, systematic review, meta-analysis, randomized controlled trials

## Abstract

**Background:**

Family members of intensive care units (ICUs) often experience psychological difficulties like anxiety and depression, which affect family functioning and their ability to support patient recovery. Digital health interventions (DHIs) offer accessible and timely support; yet, evidence regarding their effectiveness for ICU families demonstrates inconsistent.

**Objective:**

This systematic review and meta-analysis aimed to explore the impact of DHIs on psychological outcomes, quality of life (QoL), and quality of communication (QOC) among ICU family members.

**Methods:**

A total of 9 databases (CNKI [China Knowledge Resource Integrated Database], Wanfang, VIP [Weipu Database], SinoMed, Cochrane Library, PubMed, Embase, CINAHL, and Web of Science) were searched for randomized controlled trials (RCTs) published up to October 11, 2025. Eligible studies evaluated DHIs targeting adult ICU patients’ family members and reported psychological outcomes, QoL, or QOC; interventions used solely for monitoring or tracking were excluded. Furthermore, 2 reviewers independently screened studies, extracted data, and assessed the risk of bias using the Cochrane Risk of Bias Tool. Random-effects meta-analyses were performed using the Hartung-Knapp-Sidik-Jonkman method. Prediction intervals (PIs) were calculated to reflect between-study heterogeneity. Subgroup analyses were performed by patients’ primary diagnosis, relationship with patients, and type of digital health technology. Evidence quality was assessed using the Grading of Recommendations, Assessment, Development, and Evaluation (GRADE) approach.

**Results:**

In total, 17 RCTs involving 1864 participants were included. Interrater agreement was high across study selection and assessment processes (κ=0.55-1.00). Most studies were judged to have some concerns about the risk of bias. Meta-analyses showed no statistically significant pooled effects of DHIs on anxiety (standardized mean difference [SMD] –0.34, 95% CI –0.68 to 0.00; 95% PI –1.46 to 0.79; *P*=.05), depression (SMD –0.26, 95% CI –0.52 to 0.01; 95% PI –1.11 to 0.60; *P*=.06), posttraumatic stress disorder (SMD –0.21, 95% CI –0.49 to 0.06; 95% PI –1.03 to 0.60; *P*=.11), QoL (SMD 0.09, 95% CI –0.10 to 0.28; 95% PI –0.41 to 0.60; *P*=.36), or QOC (SMD 0.14, 95% CI –0.03 to 0.31; 95% PI –0.26 to 0.55; *P*=.10). Wide PIs indicated substantial variability in intervention effects across settings. The certainty of evidence ranged from very low to moderate.

**Conclusions:**

This review emphasizes significant uncertainty in estimated effects and provides the first thorough synthesis of RCT evidence on DHIs for ICU family members. The inherently high heterogeneity and low certainty of the underlying evidence limit the conclusions of this review, despite its methodological rigor and use of PIs. Compared with previous reviews, this review concentrates on the ICU family members and includes more recent research. Future studies should focus on high-risk subgroups, use mixed methods designs, and develop theory-informed, personalized, and interactive DHIs. DHIs may enable proactive, customized communication techniques for families of ICU patients, even when there are few formal clinical recommendations.

**Trial Registration:**

PROSPERO CRD420251044704; https://www.crd.york.ac.uk/PROSPERO/view/CRD420251044704

## Introduction

Patients admitted to the intensive care units (ICUs) frequently experience varying degrees of organ or system failure, which can be life-threatening [1]. Their families may endure enormous burdens and suffer from psychological disorders like anxiety, depression, and posttraumatic stress disorder (PTSD). The Society of Critical Care Medicine’s 2024 Clinical Practice Guidelines on Family-Centered Care for Adult ICUs underscore the need for delivering patient-centered and family-centered care in the ICU to uphold clinical excellence [2]. Nonetheless, various psychological challenges for family members, including anxiety, depression, acute stress disorder, PTSD, and complicated grief, are induced by the uncertainty of disease trajectories, the complexity of medical decisions, and the urgent demand for time. The term “postintensive care syndrome family” (PICS-F) encompasses these mental health disorders [3,4]. In addition to undermining family functioning and integrity, these problems may hinder the family’s ability to facilitate the patient’s recovery in the ICU [5,6].

A retrospective study [6] showed that the prevalence of psychological disorders related to PICS-F among family members of ICU patients within 6 months post admission was 15.1%, with spouses, as primary caregivers, exhibiting a heightened risk of psychological disorders. Furthermore, 1 research indicated that 10% to 69% of family members of ICU patients experienced moderate to severe psychological distress, including symptoms of anxiety, depression, and PTSD, potentially persisting for up to a year [7,8]. Family members endure not only emotional distress, such as anxiety and depression, but also are influenced by several biopsychosocial factors, including sleep deprivation, diminished quality of life (QoL) and mental health, and insufficient social support. These factors make it difficult for them to provide adequate family support to facilitate the patient’s recovery [5]. Common contributors to PICS-F among family members include female sex, relatively younger age, younger patient age, lower educational level, unmarried status, inadequate communication skills of ICU medical personnel, and the degree of family participation in decision-making [3,9]. Several strategies, such as ICU diaries [10], information support, and peer support, have been implemented to mitigate PICS-F but often focus on the patient’s hospitalization problems [11,12], and due to the family dynamics, geography, family resources, and family values, the effect of related intervention on PTSD, anxiety, and depression among family members is unclear [13]. Traditional educational tools are rarely implemented in the ICU environment due to high service costs, limited resources, and a lack of personalization [14,15]. With the rapid development of internet technology, mobile health care solutions have become popular, providing innovative psychological empowerment interventions [16].

Digital health technology (DHT) was initially introduced by the World Health Organization in the Global Strategy for Digital Health (2020-2024) and is defined as a comprehensive technical practice that facilitates clinical care, health-related education for patients and professionals, and public health through the extensive usage of electronic information and communication technologies [17,18]. Compared with conventional interventions, digital health interventions (DHIs) amalgamate computing platforms, connectivity, software, networked devices, and sensors—including smartphone apps, wearable devices, web-based communication platforms, videoconferencing, chatbots, artificial intelligence, and virtual reality (VR) technologies—leveraging technological advancements to transcend temporal and spatial limitations [19,20]. DHIs provide family members with greater accessibility, affordability, and convenience in receiving timely support and information, which is critical in the ICU context. In addition, DHIs help reduce health care spending [21,22]. DHIs have been shown to improve the psychological outcomes and QoL of caregivers, such as depressive symptoms, perceived stress, anxiety, and stress in other high-stress situations, such as caregivers of patients with cancer [23], dementia [24,25], and mental illness [15]. This study highlights the potential value of its application to family members of ICU patients. However, other studies have suggested that DHIs have little effect on alleviating the anxiety and depression of these family members [26]. Therefore, there are ambiguities in the existing research results on whether DHIs can effectively alleviate the psychological distress symptoms of family members of ICU patients [26].

After searching relevant databases, no meta-analysis of these intervention outcomes has been found. Randomized controlled trials (RCTs) continue to serve as the gold standard for delivering the most rigorous scientific findings [27]. Therefore, a systematic review and meta-analysis of RCTs focusing on the effects of DHIs on ICU family members is essential. This meta-analysis will assess the effects of DHIs on alleviating psychological symptoms, improving QoL, and enhancing communication quality among ICU family members, thereby providing critical evidence for the formulation of a comprehensive and effective psychological support program.

## Methods

### Research Design

This systematic review and meta-analysis were planned, conducted, and reported following the PRISMA (Preferred Reporting Items for Systematic Reviews and Meta-analyses) [28] (Multimedia Appendix 1). A systematic literature search, screening, study selection, data extraction, and risk-of-bias assessment were conducted according to the Cochrane methodology [29].

### Search Strategy

The search strategy was reported in accordance with the PRISMA-S (Preferred Reporting Items for Systematic Reviews and Meta-analyses literature search extension) checklist (Multimedia Appendix 2). In total, 2 investigators (Chuchu Z and WJ) independently systematically searched 9 databases, including CNKI [China Knowledge Resource Integrated Database], Wanfang, VIP (Weipu Database), SinoMed, Cochrane Library, PubMed, Embase, CINAHL, and Web of Science, from inception to October 11, 2025. The search terms included “digital health,” “intensive care units,” “family,” and “randomized controlled trial,” which were pilot-tested and validated by independent researchers. No published search filters for study design were used. To identify additional studies, we manually searched the reference lists of included articles and relevant websites, including PROSPERO clinical trial registries. Before the final data analysis, the database searches were updated. Gray literature, such as conference abstracts and academic dissertations, was excluded. The whole search strategy is available in Multimedia Appendix 3.

### Eligibility Criteria

Studies were selected based on the following inclusion criteria: (1) population: adult family members (≥18 y) of adult ICU patients (a family member could be a spouse, partner, friend, or relative [blood-related or not]); (2) interventions: all DHIs, which may include one or more methods, such as telephone, video, websites, or mobile apps, wearable devices, and others; (3) control: a comparison group that received either standard care, routine care or a blank control; (4) outcomes: the primary outcomes associated with the psychological health of family members included anxiety, depression, PTSD, and other related outcomes like QoL; and (5) study type: RCTs.

Studies were excluded if they met one of the following criteria: (1) articles were excluded if the DHI was used for monitoring or tracking purposes only; (2) duplicate publications, or (3) full text was not available.

### Study Selection

The search results were imported into NoteExpress (Aegean Software) for review. After removing duplicates, 2 investigators (Chuchu Z and RY) independently screened the titles and abstracts, then reviewed the full texts of the remaining records. Any disagreements were resolved by discussion with the third investigator (WS). The κ coefficients were used to specify the consistency of independent work. The degree of consistency was identified as slight, fair, moderate, substantial, and almost perfect when the κ coefficients were calculated as 0.00-0.20, 0.21-0.40, 0.41-0.60, 0.61-0.80, and 0.81-1.00, respectively [30].

### Data Extraction

Two authors (Chuchu Z and JY) independently extracted data from each of the included studies using a structured data extraction form, including (1) study: author, year, country, and design; (2) participants: patients’ diagnosis, sample size, female, age, relationship with patients, occupation, marital status, education level, and financial burden; (3) control; (4) experimental: intervention measurement, intervention delivery (categorized as mobile app, internet-based, and others [eg, telephone calls, VR, and emails]), intervention duration, and data collection time points; (5) outcomes and measurements; and (6) results. If necessary, the corresponding authors were contacted by email and asked for the missing information. A third reviewer (XG) verified the extracted data.

### Study Risk of Bias Assessment

The Cochrane’s risk-of-bias tool for randomized trials, version 2 (RoB 2), was used by 2 independent reviewers (JZ and ZH) to assess [31,32]. The RoB 2 tool consists of 5 domains: randomization process, deviations from intended intervention, missing outcome data, measurement of the outcome, and selection of the reported result. The risk of bias was assessed as low, some concerns, or high in the RoB 2 based on responses to signal questions in each domain. The κ coefficient was calculated for each item to identify the degree of consistency.

### Quality of Evidence

The Grading of Recommendations, Assessment, Development, and Evaluation (GRADE) approach was used to assess the certainty of the body of evidence [33]. According to the approach, the evidence quality of RCTs was initially graded as high and downgraded to moderate, low, or very low when any limitations were identified in risk of bias, inconsistency, directness, imprecision, or publication bias. However, the evidence could be upgraded by a significant effect and dose-response gradient. Furthermore, 2 authors (Chaoyi Z and YS) independently assessed the quality of evidence using the GRADE handbook, and the interrater agreement was evaluated using the κ coefficient. Disagreements were resolved through discussion, or help was sought from a third author (YZ).

### Statistical Analysis

For continuous outcomes, the Hartung-Knapp-Sidik-Jonkman random-effects method [34] was used to obtain standardized mean differences (SMDs) with 95% CIs when studies used different psychometric scales. In studies with follow-up lasting more than 1 month, data from the shortest follow-up period were used. A meta-analysis was undertaken if more than 2 studies reported outcomes in the same format; otherwise, the studies’ findings were described narratively [35]. We applied random-effects models because of suspected heterogeneity across studies to provide a relatively conservative estimate of the combined results [36]. Moreover, the prediction intervals (PI) were reported to indicate heterogeneity in the same metric as SMD and to reflect the variation in actual treatment effects expected in future studies, which aids clinical decision-making [37]. A leave-one-out sensitivity analysis was performed to assess the robustness of the synthesized results. Subgroup analysis was conducted to explore the potential sources of heterogeneity. These analyses were based on patient primary diagnosis (medical vs surgical), relationship with patients (spouse vs children), and type of DHT (education-based vs communication-based vs mental health–related). Finally, if sufficient studies were available for the outcome (n>10), a funnel plot was generated to assess potential small-study effects [38]. The Egger test was performed to further quantify funnel plot asymmetry, with a *P* value <.05 considered statistically significant [39]. A 2-tailed *P*<.05 was considered statistically significant. All analyses and visualizations were conducted using the meta package in R software (version 4.5.1; R Core Team).

### Ethical Considerations

This study and paper used published data, so no ethics approval was needed.

## Results

### Study Selection

**Figure 1:**
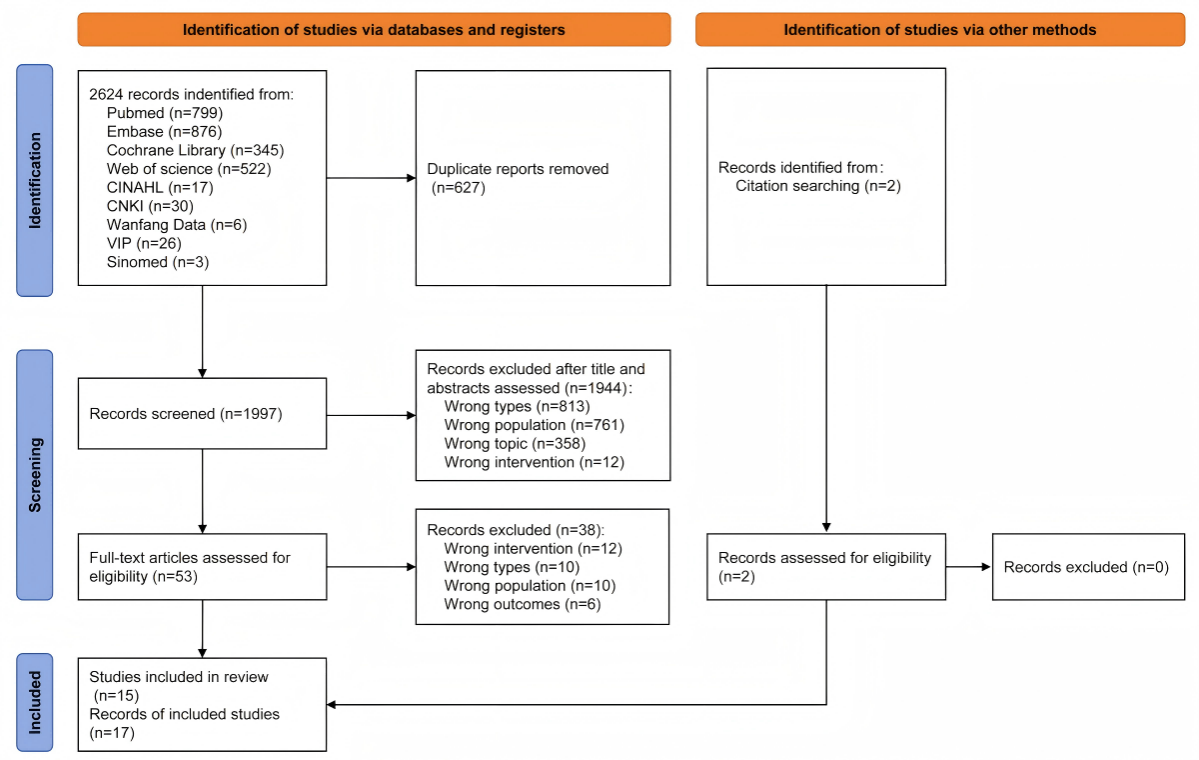
PRISMA (Preferred Reporting Items for Systematic Reviews and Meta-analyses) flow diagram. CNKI: China Knowledge Resource Integrated Database; VIP: Weipu Database.

The PRISMA flow diagram in Figure 1 summarizes the decision pathway for final study inclusion. In total, 2 authors (Chuchu Z and WJ) independently conducted the study selection process, and the κ coefficients for title or abstract screening and full-text reading were 0.82 (*P*<.001) and 0.84 (*P*<.001), respectively, indicating almost perfect consistency in these steps. We identified 2624 records through database searches and 2 records through citation searching. After title and abstract screening, we excluded 1944 records and identified 55 records for full-text evaluation. After the full-text screening, we excluded 38 records that did not meet the inclusion criteria and included 17 studies [14,16,26,40-53].

### Study Characteristics

#### Overview

The characteristics of all the included studies are summarized in Table 1. Among the 17 RCTs, 2 were cluster RCTs [41,43], and 4 were pilot RCTs [26,45,47,53]. Studies were conducted in the United States (n=8) [26,41,42,45,47,51-53], China (n=3) [16,40,50], the Netherlands (n=1) [43], Norway (n=1) [48], Germany (n=1) [44], Austria (n=1) [14], Iran (n=1) [46], and Korea (n=1) [49], which were published from 2017 to 2025.

**Table 1:** Characteristics of the studies and participants in the included studies.

Study (year, country)	Design	Patient’s diagnosis	Sample size, n (I/C^a^), % of female	Age (y), mean (SD)	Relationship with patients, n (%)	Occupation, n (%)	Marital status, n (%)	Education level, n (%)	Financial burden, n (%)
Carlozzi et al [51] (2025, United States)	RCT^b^	Traumatic brain injury 100%	257 (129/128), 79.1	52.25 (14.48)	Partner 104 (40.9) Parent 83 (32.7) Child 36 (14.2) Sibling 20 (7.9) Other 6 (2.4)	Employed 157 (61.8) Retired 58 (22.8) Unemployed 15 (5.9) Disabled 8 (3.1) Other 16 (6.3)	—^c^	—	—
Chiang et al [40] (2017, China)	RCT	—	74 (39/35), 62.2	—	Children 32 (43.2) Parent 21 (28.4) Spouse 9 (12.2) Sibling 5 (6.8) Other 7 (9.5)	—	Married 45 (60.8) Single 22 (29.7) Divorced 7 (9.5)	Below university 46 (62.2) University or above 26 (35.1)	High 32 (43.2) Low 31 (41.9)
Cox et al [42] (2019, United States)	RCT	Medical 50.9% Neurological 22% Surgical 13.4% Trauma 11.9% Cardiology 1.8%	275 (137/138), 73.1	51.20 (12.60)	Spouse 128 (46.5) Children 65 (23.6) Parent 54 (19.6) Sibling 22 (8) Other 6 (2.2)	Employed 184 (66.9) Retired 54 (19.6) Disabled 18 (6.5) Unemployed 17 (6.2) Other 2 (0.7)	Married 210 (76.4) Single 28 (10.2) Divorced 37 (13.4)	Below university 157 (57.1) University or above 118 (42.9)	Low 130 (42.3) No 80 (29.1) Moderate 45 (16.4) High 20 (7.3)
Cox et al [41] (2024, United States)	Cluster RCT	Medical 51.6% Neurological 22% Shock 14% Trauma 13%	111 (55/56), 83	51.00 (15.00)	Spouse 50 (45) Parent 23 (20.7) Children 22 (19.8) Sibling 11 (9.9) Other 5 (4.5)	Employed 81 (73) Retired 19 (17.1) Disabled 7 (6.3) Unemployed 4 (3.6)	Married 67 (60.4) Single 21 (18.9) Divorced 23 (20.7)	Below university 20 (18) University or above 91 (82)	No 45 (40.5) Low 44 (39.6) Moderate 13 (11.7) High 9 (8.1)
Cox et al [52] (2025, United States)	RCT	Medical 37.1% Neurological 27.8% Cardiac 24.5% Surgical 8% Community medical-surgical 2.7%	151 (76/75), 72.9	57.40 (12.90)	Spouse 80 (60.6) Child 36 (27.3) Parent 12 (9.1) Sibling 2 (1.5) Other 2 (1.5)	Employed 82 (62.1) Retired 36 (27.3) Disabled 11 (8.3) Unemployed 3 (2.3)	Married 107 (81.1) Single 13 (9.9) Divorced 12 (9.1)	—	—
Yuan et al [50] (2023, China)	RCT	Surgical 100%	98 (50/48)	—	Children 66 (67.3) Spouse 24 (24.5)	—	—	—	—
Drop et al [43] (2025, Netherlands)	Cluster RCT	Medical 60.9% Surgical 31.7% Trauma 7.5%	189 (100/89), 53.4	47.35 (12.36)	Children 71 (37.6) Spouse 62 (32.8) Sibling 11 (5.8) Parent 9 (4.8) Other 7 (3.7)	—	—	Below university 142 (75.1) University or above 18 (9.5)	—
Gawlytta et al [44] (2022, Germany)	RCT	Severe sepsis 100%	25 (12/13), 64	54.00 (11.01)	Spouse 25 (100)	—	Married 21 (84)	Below university 15 (60) University or above 10 (40)	—
Hoffmann et al [14] (2023, Austria)	RCT	Nonsurgical 69.7% Surgical 30.3%	89 (46/43), 66.3	47.00 (13.00)	Spouse 36 (40.4) Children 31 (34.8) Sibling 9 (10.1) Parent 8 (9) Other 5 (5.6)	Employed 67 (75.3) Unemployed 22 (24.7)	—	Below university 55 (61.8) University or above 34 (38.2)	—
Petrinec et al [45] (2023, United States)	Pilot RCT	Infectious disease 31.7% Neurological 13.3% Cardiovascular 11.7% Gastrointestinal 8.3% Trauma 8.3%	60 (30/30), 61.7	46.39 (13.04)	Children 23 (38.3) Spouse 16 (26.7) Parent 8 (13.3) Sibling 7 (11.7) Other 6 (10)	—	—	—	—
Shariati et al [46] (2021, Iran)	RCT	Patient with COVID-19	67 (33/34), 46.3	38.68 (11.18)	Children 37 (55.2) Spouse 21 (31.3) Parent 4 (6) Sibling 1 (1.5) Other 4 (6)	Employed 62 (92.5) Retired 2 (3) Student 3 (4.5)	Married 41 (61.2) Single 26 (38.8)	Below university 4 (6) University or above 63 (94)	—
Shin et al [26] (2025, United States)	Pilot RCT	Respiratory 42.9% Cardiovascular 21.4% Sepsis 17.9% Surgical 17.9%	28 (14/14), 85.7	50.93 (16.05)	Spouse 19 (67.9) Parent 4 (14.3) Sibling 3 (10.7) Children 2 (7.1)	Employed 12 (42.9) Unemployed 6 (21.4) Retired 8 (28.6) Disabled 2 (7.1)	—	Below university 14 (50) University or above 14 (50)	—
Suen et al [47] (2021, United States)	Pilot RCT	Medical 100%	48 (23/25), 62.5	56.37 (12.95)	Spouse 16 (33.3) Children 16 (33.3) Sibling 4 (8.3) Parent 4 (8.3) Other 8 (16.7)	—	—	Below university 24 (50) University or above 24 (50)	—
Vranceanu et al [53] (2020, United States)	Pilot RCT	Neurological 100%	58 (29/29), 67.2	52.25 (14.48)	Spouse 46 (79.3) Parent 7 (12.1) Sibling 2 (3.4) Other 3 (5.2)	Employed 41 (70.7) Retired 10 (17.2) Other 7 (12.1)	—	Below university 29 (50) University or above 29 (50)	—
Watland et al [48] (2025, Norway)	RCT	Respiratory 37.7% Cardiovascular 20.4% Accident 18.4% Neurological 13.3% Sepsis 12.7%	196 (101/95), 64.8	46.80 (13.51)	Children 79 (40.3) Spouse 45 (23) Parent 32 (16.3) Sibling 26 (13.3) Other 14 (7.1)	—	—	Below university 73 (37.2) University or above 123 (62.8)	—
Woo et al [49] (2024, Korea)	RCT	Surgery 71.1% Shock 18.4% Multiple traumas 7.9% Respiratory 2.6%	38 (18/20), 68.4	51.60 (11.80)	Spouse 20 (52.6) Children 8 (21.1) Parent 5 (13.2) Sibling 4 (10.5) Other 1 (2.6)	—	—	—	—
Xiong et al [16] (2025, China)	RCT	Patients who underwent HVR^d^	100 (50/50), 61	—	Children 48 (48) Spouse 42 (42) Parent 8 (8) Sibling 1 (1) Other 1 (1)	Employed 92 (92) Retired 8 (8)	Married 93 (93) Not married 7 (7)	Below university 75 (75) University or above 25 (25)	Moderate 53 (53) Low 41 (41) High 6 (6)

^a^I/C: intervention/control.

^b^RCT: randomized controlled trial.

^c^Not applicable.

^d^HVR: heart valve replacement.

#### Participants

The sample sizes ranged from 25 to 275, yielding a total of 1864 participants (intervention: 942, 50.5% and control: 922, 49.5%). Regarding patient types, only 1 study [40] did not report; 11 studies [14,26,41-48,52] focused on medical patients; and 5 studies [16,49-51,53] focused on surgical patients. Furthermore, 7 studies [41,42,44,49,50,52,53] recruited not only family members but also ICU patients or ICU health care staff, whereas the remaining 10 studies recruited only family members. In addition, 14 studies reported the mean ages of family members, ranging from 38.68 (SD 11.18) years to 57.40 (SD 12.90) years. Except for the study by Yuan et al [50], all studies reported that 46.3% to 85.7% of participants were female. Concerning the relationship to patients, 7 studies [16,40,43,45,46,48,50] reported that the main participants were children, and 10 studies reported that they were spouses. Moreover, 9 studies [14,16,26,41,42,46,51-53] reported participants’ occupation, indicating most of them were employed; 10 studies [14,26,43,45,47-51,53] did not report marital status; the other 7 did, and the majority of participants were married. Regarding educational level, 5 studies [45,49-52] did not report it; 8 reported that most participants had nontertiary education; and 4 reported that most participants had university education or higher. Financial burden was not reported in 13 studies; 2 studies [41,42] reported no or low burden, and 2 studies [16,40] reported moderate or high burden (Table 1).

#### Intervention

In the included studies, various types of DHT were used. In total, 9 studies [16,26,40,41,45,49-53] used mobile apps, 5 studies [14,42,44,46,47] used internet-based technology, and 3 used other technologies (1 was a video tool [49], 1 was a digital assessment tool [48], and 1 was VR technology [43]). Furthermore, 10 studies [41-46,49,51-53] reported the specific duration of the intervention, and the remaining studies indicated that family members could use digital intervention tools during the ICU or within months after discharge. Regarding data collection timing, in addition to pre- and postintervention assessments, 6 studies [14,16,26,43,45,51] collected data at 1 month post intervention; 1 study [45] at 2 months post enrollment; 10 studies [14,26,41-43,47,48,51-53] at 3 months post enrollment or discharge; 4 studies [26,42,43,51] at 6 months post discharge; and 2 studies [14,51] at 12 months post admission. No adverse outcomes or events were reported across studies (Table 2).

**Table 2:** Characteristics of the outcomes and measurements in the included studies.

Study (year, country)	Control	Experimental				Outcomes and measurements	Results
		Intervention measurement	Intervention delivery	Intervention duration	Data collection time points		
Carlozzi et al [51] (2025, United States)	Self-monitoring only	Self-monitoring plus personalized self-care from the CareQOL app	Mobile app	10-day run-in period followed by a 6-month home monitoring period	baseline; 1-12 months	Caregiver appraisal: CAS^a^ Adaptability: MPAI-4^b^ PTSD^c^: PCL-5^d^ Caregiver-specific anxiety: PROMIS^e^ Sleep-related impairment: PROMIS Sepression: PROMIS Fatigue: PROMIS Perceived stress: PROMIS Self-efficacy general: PROMIS Overall health: PROMIS	Although we did not observe improvements in HRQOL^f^, physical activity, or sleep, we found that across all measures, approximately one-third of participants showed clinically meaningful improvements, one-third remained the same, and one-third worsened. Care partners who reported engaging in the intervention were more likely to show improvement than those who did not. There was preliminary support for factors that being male, caring for a person with posttraumatic stress symptoms, living in the same household as the person with TBI^g^, being a spousal care partner, working, and being diagnosed with COVID-19 during the study were associated with increased risk for adverse outcomes.
Chiang et al [40] (2017, China)	EF-R^h^	EF-T^i^	Interactive mobile technology	During ICU^j^	Baselines; the posttest one day after EF-T or EF-R	Depression: C-DASS^k^ Anxiety: C-DASS Stress: C-DASS Satisfaction: CPCS^l^	The EF-R group had a slightly lower stress score than the EF-T group at posttest, but the difference was statistically insignificant. There was also no significant improvement in anxiety after EF-T. The EF-T group had a significantly better reduction of depression score than the EF-R group. Information need satisfaction was not significantly different between intervention and control groups.
Cox et al [42] (2019, United States)	Usual care practices followed by a family meeting	A web-based decision aid	Web-based	2 days	In person at enrollment but before randomization; on study day 3; via telephone at 3 months and 6 months	Clinician surrogate concordance: CSCS^m^ Comprehension of diagnosis, treatment, and prognosis: MCS^n^ Clinician communication satisfaction: QOC^o^ Anxiety: HADS^p^ Depression: HADS PTSD: PTSS^q^ Decision-making uncertainty: DCS^r^ Patient-centeredness of care: PPPC^s^	Concordance improvement did not differ between intervention and control groups. Intervention surrogates had a greater reduction in decisional conflict than control surrogates. Intervention and control primary surrogates did not differ in medical comprehension or communication. Surrogates’ total scores on anxiety, depression, and PTSD also did not differ at 3 and 6 months.
Cox et al [41] (2024, United States)	Usual ICU care without protocolized family meetings	ICUconnect uses text messages and emails	Mobile app	7-10 days	Baselines; study days 1, 3, and 7; 3 months	Needs at the End-of-Life Screening Tool: NEST^t^ Goal concordance of care Quality of communication: QOC Patient-centeredness of decision-making, eliciting concerns, discrimination: IPC^u^ Depression: PHQ-9^v^ Anxiety: GAD-7^w^ PTSD: PTSS	There was greater improvement in NEST scores among intervention recipients between baseline and both day 3 and day 7. There were no treatment group differences at 3 months in psychological distress symptoms. Change from baseline to study day 3 was not statistically significantly different between intervention and control groups in IPC-Elicited Concerns, and IPC-Decision Making subscales, as well as QOC scores. Although intervention family members reported a numerically greater improvement in goal-concordant care compared with control family members, this finding was not statistically significant. White family members experienced a greater reduction in NEST scores compared with Black family members at day 3 and day 7.
		Intervention measurement	Intervention delivery	Intervention duration	Data collection time points		
Cox et al [52] (2025, United States)	Usual ICU care	PC planner	Mobile app-based platform	7 days	Baseline; day1; day3; day 7; and 3 month	Needs at the End-of-Life: NEST Goal concordant of care: GCC^x^ Quality of communication: QOC Patient-centeredness: PPPC Depression: PHQ-9 Anxiety: GAD-7 PTSD: PTSS	Treatment group differences in estimated mean NEST scores were similar at 3 days and at 7 days. Goal concordance, communication quality, and psychological distress symptoms did not differ. 29 (38.2%) intervention participants had palliative care consultations, compared with only 3 (4%) among controls (P<.001).
Yuan et al [50] (2023, China)	Routine care	Video visitation	WeChat video call	During the ICU, every afternoon	Admission to the ICU, and discharge from the ICU	Anxiety: SAS^y^ Satisfaction: questionnaire in the hospital	There were no statistically significant differences between the groups in patients’ anxiety and depression scores, and no statistically significant differences in delirium incidence between the groups. There were no significant differences in changes in family members’ anxiety scores. A statistically significant difference in satisfaction was found between the two groups of patients, and the result of family members’ satisfaction was also statistically significant.
Drop et al [43] (2025, Netherlands)	Standard care	Standard care plus ICU-VR^z^	VR	14 min	Immediately after enrollment at ICU; admission at ICU; discharge at 1, 3, and 6 months post-ICU discharge	PTSD: IES-R^aa^ Anxiety: HADS Depression: HADS Quality of life: SF-36^ab^ Relatives’ understanding: CQI^ac^-relatives ICU Perceived stress and appreciation of ICU-VR: family satisfaction with ICU care tools	Relatives who received ICU-VR did not experience a decrease in PTSD, anxiety, or depression. There was no significant difference between the median mental quality of life, physical quality of life, or understanding of ICU care. Patients in the intervention group highly endorsed ICU-VR, favoring it over traditional informational brochures, and the majority stated it improved their understanding of ICU treatment.
Gawlytta et al [44] (2022, Germany)	5-week waiting period	iCBT^ad^: 10 writing assignments	Internet-based	Two 50-minute sessions per week over 5 weeks	Start of intervention or waiting; end of intervention or waiting; end of intervention in WL^ae^ control group	PTSD: PCL-5 Depression: BSI-18^af^ Anxiety: BSI-18 Somatization: BSI-18 Relationship satisfaction: RAS^ag^ Health-related quality of life: EQ-5D-5L (five-level EuroQol five-dimensional questionnaire)	There was no evidence of a difference in PCL-5 pre-post change between iCBT and WL. iCBT led to a larger RAS change than waiting, with effects in favor of waiting. For all other secondary efficacy outcomes, we did not observe evidence for an association between score changes and iCBT in the primary analysis set.
Hoffmann et al [14] (2023, Austria)	Control website	Intervention website	Online	Not reported	Within 2 days of the patient’s admission to the ICU; 30 days after T0; 90, and 365 days after admission	PTSD: IES Anxiety: HADS Depression: HADS Frequency and times of website use	An informative website did not result in reduced PTSD and depression symptoms.
Petrinec et al [45] (2023, United States)	Usual care	A mental health app (Sanvello)	Smartphone app	Each module contains 6 to 9 lessons (15 min per lesson)	Upon enrollment (within 5 days of ICU admission); 30 and 60 days after enrollment	Anxiety: HADS Depression: HADS PTSD: PCL-5 Quality of life: SF-12^ah^ self-efficacy: MHSES^ai^ Use of the app Satisfaction with the app Selection of the app	Anxiety and depression symptom severity decreased significantly over time in the intervention group but not in the control group. Family members logged in to the app a mean of 11.4 times and spent a mean of 50.16 minutes using the app. Participants’ mean rating of their satisfaction with the mental health app was 4.19 out of 5.
Shariati et al [46] (2021, Iran)	All common interventions except web-based communication	Web-based communication, audio or video (WhatsApp)	Web-based	4 consecutive days for 10 to 15 minutes per day	Before intervention; within 24 hours after the intervention in both groups	Stress: PSS^aj^	After the intervention, the mean PSS-14 in the intervention group was significantly lower than that of the control group.
Shin et al [26] (2025, United States)	Standard Android tablet loaded with MyChart	The tablet also contained the MyChart Bedside app and selected games as standard in the hospital	Tablet computer app	During ICU	Baseline; extubation or ICU discharge; 1, 3, and 6 months follow-up	Anxiety: HADS Depression: HADS PTSD: IES-R Communication difficulty: FCS^ak^	No statistically significant difference was found between groups in changes in family psychological outcomes. The VidaTalk was associated with a slight to medium improvement in anxiety symptoms at 1 month. The VidaTalk group had lower PTSD-related symptoms than the AC^al^ group with a medium effect size at 1 month and a medium-to-large effect size at 3 months. Compared with the perceived communication difficulty, although not statistically significant, the mean change in FCS score was larger for the intervention group than for AC, with a small effect size.
Suen et al [47] (2021, United States)	iPad without the tool	iPad preloaded with the Family Support Tool	Interactive website	During ICU	Baseline; 48 h of study enrollment; 5-9 days of study enrollment; 3 months	Tool usability and acceptability Perceived effectiveness: internally generated 11-item questionnaire Quality of communication: QOC	Surrogates reported that the tool was highly usable, acceptable, and effective. Surrogates who used the tool reported higher overall quality of communication and higher quality in shared decision-making, but the difference did not reach statistical significance.
Vranceanu et al [53] (2020, United States)	Mimic the dose and duration of RT^am^ without teaching RT skills	RT is an active intervention. The first 2 sessions were taught at the bedside, and the next 4 were taught after discharge	Web-based app on a smartphone	30 minutes per session	Baseline; 6 weeks; and 12 weeks	Feasibility of recruitment and intervention delivery, credibility, and satisfaction Depression and anxiety: HADS PTSD: PCL-C Mindfulness: CAMS-R^an^ Coping: MOCS-A^ao^ Dyadic interactions: DRS^ap^	Feasibility, adherence, fidelity, satisfaction, credibility, and expectancy exceeded benchmarks set a priori. Participation in RT was associated with statistically and clinically significant improvement between baseline and postintervention in symptoms of depression, anxiety, and PTSD. Improvements sustained through the 12-week follow-up visit. RT-dependent improvement in dyadic interpersonal interactions for survivors.
Watland et al [48] (2025, Norway)	Standard care	The Caregiver Pathway 4-step model	Digital assessment tool	From admission to 3 months after discharge	Baseline; 3 months	PTSD: IES-R Anxiety: HADS Depression: HADS Quality of life: SF-12 Self-efficacy: GSE^aq^ Hope: Herth Hope Index	The Caregiver Pathway is associated with reduced symptoms of PICS-F^ar^ in terms of reduced symptoms of PTSD, anxiety, and depression for caregivers of surviving patients.
Woo et al [49] (2024, Korea)	Daily phone calls	Daily virtual visits	Zoom app	Began on the first day after ICU admission until ICU discharge, lasting for a maximum of 7 days (each limited to 15 min)	At study enrollment after ICU admission; at the end of the study	Satisfaction concerning ICU care and decision-making processes: FS-ICU 24^as^ Anxiety: HADS Depression: HADS	The FS-ICU 24 survey score was significantly higher in the virtual visitation group. The reduction in HADS-A and HADS-D scores between the 2 time points was significantly larger in the virtual visitation group.
Xiong et al [16] (2025, China)	Standard care	Wab-WPPEP	WeChat applet	Patient’s admission to the ICU until 1 month after discharge	Preoperative baseline; before transfer from the ICU; before discharge from the hospital; 1 month after discharge	Anxiety: HADS Depression: HADS PTSD: IES-R Quality of life: SF-36	The intervention group exhibited greater improvements in anxiety, depression, and quality of life compared with the control group. PTSD scores were also significantly lower in the intervention group.

^a^CAS: caregiver appraisal scale.

^b^MPAI-4: Mayo-Portland Adaptability Inventory-Fourth Edition.

^c^PTSD: posttraumatic stress disorder.

^d^PCL-5: PTSD (posttraumatic stress disorder) Checklist for DSM-5 (Diagnostic and Statistical Manual of Mental Disorders, [Fifth Edition]).

^e^PROMIS: Patient-Reported Outcomes Measurement Information System.

^f^HRQOL: health-related quality of life.

^g^TBI: traumatic brain injury.

^h^EF-R: education of families by routine.

^i^EF-T: education of families by interactive tabs.

^j^ICU: intensive care unit.

^k^C-DASS: Depression Anxiety Stress Scale-Chinese.

^l^CPCS: Society of Critical Care Medicine’s Family Needs Assessment Questionnaire’s Communication and Physical Comfort Scale.

^m^CSCS: clinician-surrogate concordance scale.

^n^MCS: medical comprehension scale.

^o^QOC: quality of communication questionnaire.

^p^HADS: Hospital Anxiety And Depression Scale.

^q^PTSS: posttraumatic stress symptom inventory.

^r^DCS: decision conflict scale.

^s^PPPC: Patient-Perceived Patient-Centeredness scale.

^t^NEST: Needs at the End-of-Life Screening Tool.

^u^IPC: interpersonal processes of care scales.

^v^PHQ-9: patient health questionnaire-9 scale.

^w^GAD-7: generalized anxiety disorder 7 scale.

^x^GCC: goal concordant of care.

^y^SAS: Self-Rating Anxiety Scale.

^z^VR: virtual reality.

^aa^IES-R: Impact of Event Scale-revised.

^ab^SF-36: 36-Item Short-Form Health Survey.

^ac^CQI: Consumer Quality Index.

^ad^iCBT: internet-based cognitive behavioral writing therapy.

^ae^WL: wait list.

^af^BSI-18: Brief Symptom Inventory-18.

^ag^RAS: Relationship Satisfaction Scale.

^ah^SF-12: 12-Item Short-Form General Health Survey.

^ai^MHSES: Mental Health Self-Efficacy Scale.

^aj^PSS: Perceived Stress Scale.

^ak^FCS: Family Communication Scale.

^al^AC: attention control.

^am^RT: Recovering Together.

^an^CAMS-R: Cognitive and Affective Mindfulness Scale-Revised.

^ao^MOCS-A: Measure of Current Status-Part A.

^ap^DRS: Dyadic Relationship Scale.

^aq^GSE: General Self-Efficacy Scale.

^ar^PICS-F: Post-Intensive Care Syndrome Family.

^as^FS-ICU: Family Satisfaction-Intensive Care Unit Questionnaire.

#### Control

Control groups varied in different studies; 12 studies [16,40-43,45-50,52] adapted usual or routine care, including regular education on the ICU environment, basic intensive care processes, the use of monitoring devices, ICU rules, spontaneous conversations with the ICU nurses and meetings with the physicians, effective communication with critically ill patients, and basic principles of patient care. In addition, 4 studies [14,26,51,53] used a standard app or website that provided only basic information, and 1 study [44] was a blank control (Table 2).

#### Outcomes and Assessment Tools

Table 2 summarizes the outcomes and measurement tools of each study. All the assessment scales used in the outcome measurement were validated. In total, 5 scales were used to assess the anxiety and depression levels of ICU patients’ families. Of the total, 9 of the included studies [14,16,26,42,43,45,48,49,53] adopted the Hospital Anxiety and Depression Scale to assess anxiety and depression. Moreover, 2 studies [41,52] assessed anxiety using the Generalized Anxiety Disorder 7 scale and depression using the Patient Health Questionnaire-9 Scale. Another study [40] assessed anxiety and depression using the Depression Anxiety Stress Scale-Chinese. One study [51] used the Patient-Reported Outcomes Measurement Information System questionnaire. One study [50] used the Self-Rating Anxiety Scale to assess anxiety. One study [44] used the Brief Symptom Inventory-18. The measurement tools used for PTSD varied among studies. In total, 3 studies [41,42,52] measured the Post-Traumatic Stress Symptom Inventory, 3 studies [44,45,53] used the PTSD Checklist for *DSM-5* (Diagnostic and Statistical Manual of Mental Disorders [Fifth Edition]), and 5 studies [14,16,26,43,48] used the Impact of Event Scale-Revised. In addition, 5 studies measured QoL with 3 scales. 1 study [44] measured the Health Questionnaire of the EQ-5D-5L; 2 studies [45,48] measured the 12-Item Short-Form Health Survey, and 2 studies [16,43] measured the 36-Item Short-Form Health Survey.

### Risk of Bias

**Figure 2:**
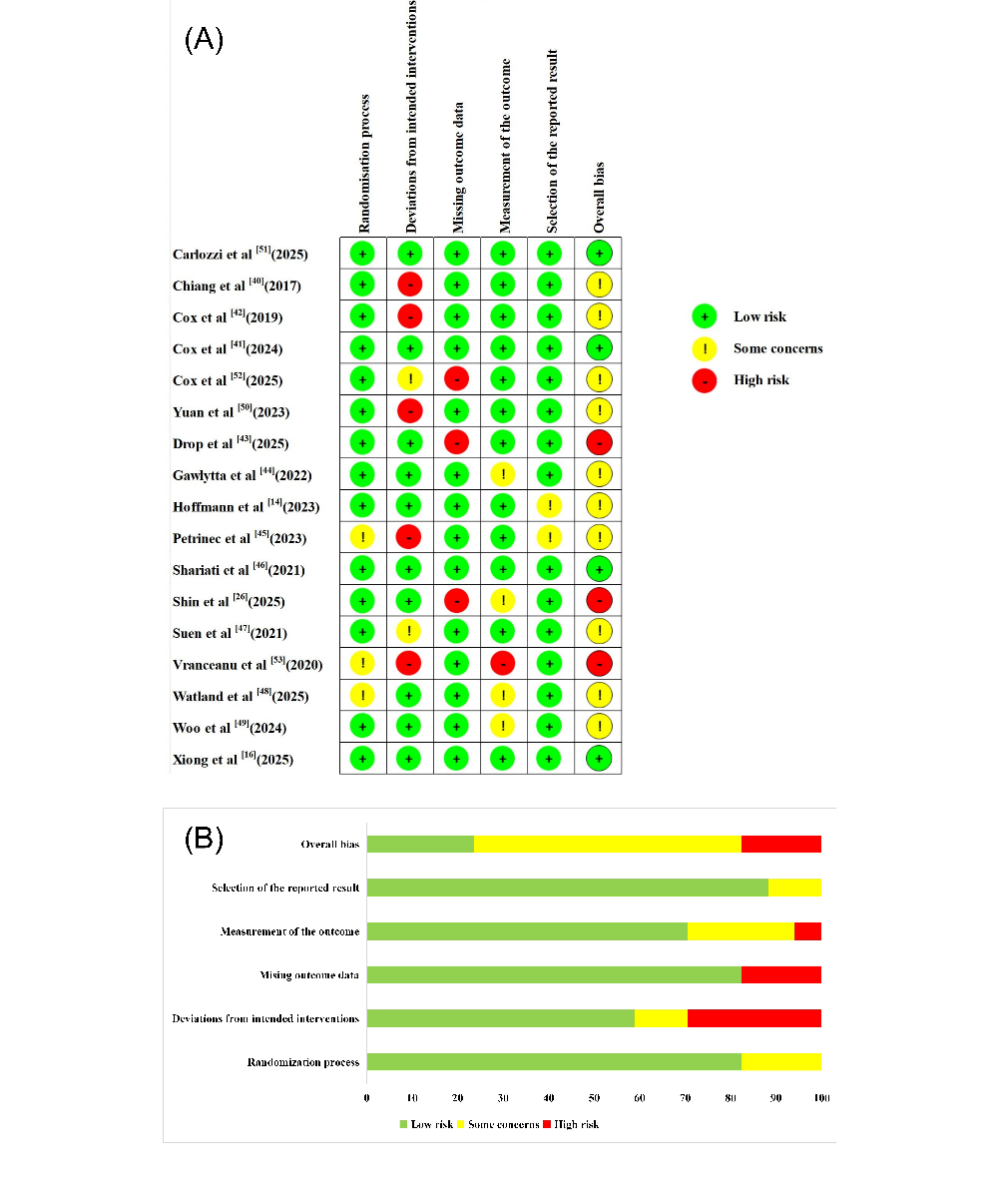
The results of the risk of bias assessment of included studies. (A) Risk of bias summary, (B) risk of bias graph [14,16,26,40-53].

Two authors (JZ and ZH) used the Cochrane Risk of Bias Assessment Tool version 2 independently to assess the methodological quality of the included studies, and the weighted κ coefficients of domains ranged between 0.55 (*P*=.002) and 1.00 (*P*<.001), indicating substantial to almost perfect interrater agreement of the independent work in this part. The results are presented in Figure 2 [14,16,26,40-53]. Specifically, all included studies were reported as randomized, but selective bias was evident because 3 studies [45,48,53] did not report allocation concealment. Due to the nature of DHIs, blinding of participants and investigators was not possible; only 1 study [14] used a double-blind design for the intervention because the structure was the same across both websites. However, almost all studies reported using intention-to-treat analysis to estimate the effect of intervention assignment, thereby reducing bias. A total of 5 studies [40,42,45,50,53] reported deviations from the intended intervention arising from the trial context, and are therefore rated as high-bias. In addition, 3 studies [26,43,52] reported incomplete data, which increased the risk of attrition bias. Furthermore, 12 studies were blinded to outcome assessors, and the other 5 studies [26,44,48,49,53] addressed bias in outcome measurement. Moreover, 2 studies [14,45] provided insufficient information to permit judgment and might have led to overestimated effects and introduced selection bias into the reported results. Finally, 4 studies were assessed as “low-risk bias,” 10 as “some concerns,” and 3 as “high-risk bias.” However, in accordance with the consensus agreement of all the authors, the studies with a high risk of overall bias were ultimately reserved.

### Quality of Evidence

The GRADE approach [33] was used to assess the overall certainty of the evidence. The κ coefficient in this part was 0.88 (*P*<.001), indicating almost perfect interrater consistency. Among the 7 outcomes analyzed, the certainty of evidence was downgraded to moderate for QoL and quality of communication (QOC). The certainty for anxiety, depression, PTSD, and self-efficacy was downgraded to low. Stress was downgraded to very low certainty of evidence (Table 3).

**Table 3:** GRADE^a^ evidence profile for digital health intervention compared with standard care for intensive care unit family members.

Outcomes	Follow-up			Certainty of the evidence^b^ (GRADE)		Relative effect (95% CI)^c^		Anticipated absolute effects	
	Participants^d^, n	Studies, n					Risk with standard care		Risk difference with digital health intervention
Anxiety	1209	12 RCTs^e^	⨁⨁◯◯ Low^f,g,h^		—^i^		—		SMD^j^ 0.34 SD lower (0.68 lower to 0)
Depression	1181	12 RCTs	⨁⨁◯◯ Low^f,g,h^		—		—		SMD 0.26 SD lower (0.52 lower to 0.01 higher)
PTSD^k^	1077	11 RCTs	⨁⨁◯◯ Low^f,g,h^		—		—		SMD 0.21 SD lower (0.49 lower to 0.06 higher)
Stress	141	2 RCTs	⨁◯◯◯ Very low^g,h,l,m^		—		—		SMD 1.17 lower (3.53 lower to 1.19 higher)
Self-efficacy	204	2 RCTs	⨁⨁◯◯ Lowf,h,n,o		—		—		SMD 0.01 lower (0.28 lower to 0.27 higher)
QoL^p^	424	5 RCTs	⨁⨁⨁◯ Moderate^f,h,n^		—		—		SMD 0.09 SD higher (0.17 lower to 0.35 higher)
Quality of communication	555	4 RCTs	⨁⨁⨁◯ Moderate^f,h,n^		—		—		SMD 0.15 SD higher (0.06 lower to 0.35 higher)

^a^GRADE: Grading of Recommendations, Assessment, Development, and Evaluation.

^b^High certainty (we are very confident that the true effect lies close to that of the estimate of the effect); moderate certainty (we are moderately confident in the effect estimate: the true effect is likely to be close to the estimate of the effect, but there is a possibility that it is substantially different); low certainty (our confidence in the effect estimate is limited: the true effect may be substantially different from the estimate of the effect); and very low certainty (we have very little confidence in the effect estimate: the true effect is likely to be substantially different from the estimate of effect).

^c^The risk in the intervention group (and its 95% CI) is based on the assumed risk in the comparison group and the relative effect of the intervention (and its 95% CI).

^d^Patient or population: intensive care unit family members; setting: intensive care unit; intervention: digital health intervention; and comparison: standard care.

^e^RCT: randomized controlled trial.

^f^Most of the included studies were assessed as some concerns or high-risk bias.

^g^*I*^2^≥50%.

^h^Direct participants, interventions and outcomes.

^i^Not applicable.

^j^SMD: standardized mean difference.

^k^PTSD: posttraumatic stress disorder.

^l^Most of the included studies were assessed as low-risk or some concerns bias.

^m^Sample size is small and CI was wide.

^n^*I*^2^<50%.

^o^CI was wide.

^p^QoL: quality of life.

### Effect of DHIs

#### Psychological Outcomes

**Figure 3:**
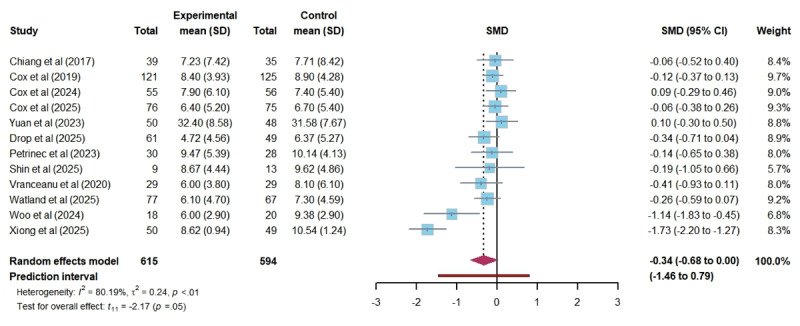
Forest plot of the effectiveness of digital health technology on anxiety. SMD: standardized mean difference [16,26,40-43,45,48-50,52,53].

**Figure 4:**
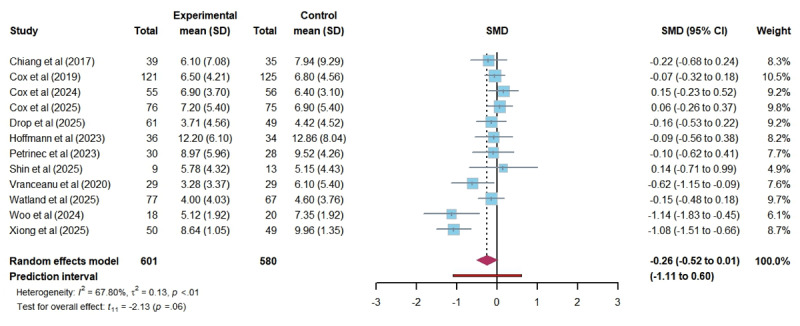
Forest plot of the effectiveness of digital health technology on depression. SMD: standardized mean difference [14,16,26,40-43,45,48,49,52,53].

**Figure 5:**
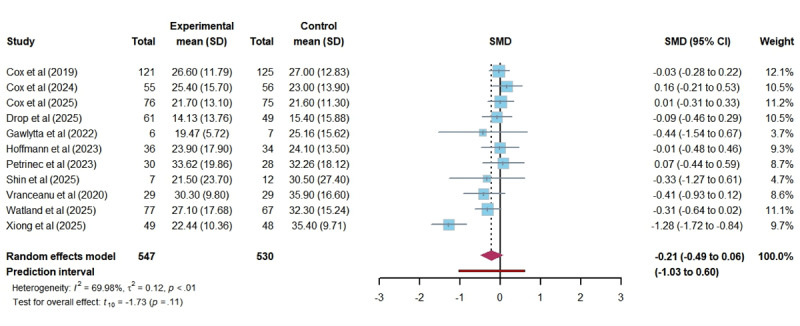
Forest plot of the effectiveness of digital health technology on post-traumatic stress disorder. SMD: standardized mean difference [14,16,26,41-45,48,52,53].

In total, 12 studies assessed the impact of DHIs on anxiety among ICU family members. The pooled results showed that DHIs did not lead to a statistically significant reduction in anxiety levels (SMD –0.34, 95% CI –0.68 to 0.00; *P*=.05). The heterogeneity among studies was substantial (*I*^2^=80.19%; τ=0.49; τ^2^=0.24), and the certainty of the evidence was rated as low. The 95% PI of the effect of DHIs on anxiety was –1.46 to 0.79, which indicates that we can be 95% confident that the true effect size from a future individual study would fall within this range (Figure 3 [16,26,40-43,45,48-50,52,53]). Similarly, 12 studies investigated the impact of DHIs on depression among ICU family members. The results revealed no significant effect (SMD –0.26, 95% CI –0.52 to 0.01; *P*=.06), with moderate heterogeneity (*I*^2^=67.80%; τ=0.37; τ^2^=0.13). The certainty of this evidence was also rated as low. A wide 95% PI (–1.11 to 0.60) suggests that future studies could show effects ranging from a large reduction to a modest increase in depression levels (Figure 4 [14,16,26,40-43,45,48,49,52,53]). In total, 11 studies evaluated the effects of DHIs on PTSD among ICU family members. The meta-analysis demonstrated no significant difference in PTSD scores between groups (SMD –0.21, 95% CI –0.49 to 0.06; *P*=.11), with substantial heterogeneity (*I*^2^=69.98%; τ=0.34; τ^2^=0.12). The certainty of this evidence was also rated as low. The 95% PI (–1.03 to 0.60) indicates that the actual effect of DHIs on PTSD could vary widely, indicating potential for both beneficial and harmful effects (Figure 5 [14,16,26,41-45,48,52,53]). Furthermore, regarding stress and self-efficacy, which were evaluated in only 2 studies each, we discussed them qualitatively. 2 studies reported different results regarding stress: 1 study [40] found no effect of DHI on stress. In contrast, another [46] reported a significant effect of DHI in reducing stress among ICU family members. Neither study [45,48] on self-efficacy found a significant benefit from DHIs. The certainty of the 2 pieces of evidence was rated as very low and low.

#### QoL

**Figure 6:**
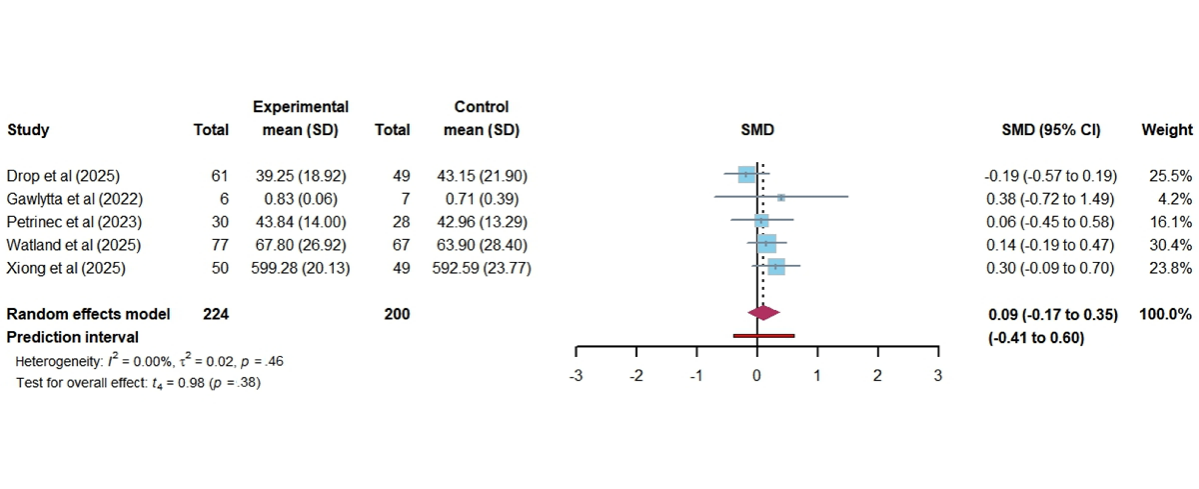
Forest plot of the effectiveness of digital health technology on quality of life. SMD: standardized mean difference [16,43-45,48].

In total, 5 studies assessed the impact of DHIs on QoL among ICU family members. A random-effect meta-analysis indicated no statistically significant improvement in QoL (SMD 0.09, 95% CI –0.10 to 0.28; *P*=.36). Despite the absence of heterogeneity (*I*^2^=0.00%; τ=0.14; τ^2^=0.02), the actual effect of DHIs on QoL in the future falls within the broader PI range (–0.41 to 0.60), suggesting that future outcome could vary significantly across different populations and settings (Figure 6 [16,43-45,48]). The certainty of this evidence was rated as moderate.

#### QOC

**Figure 7:**
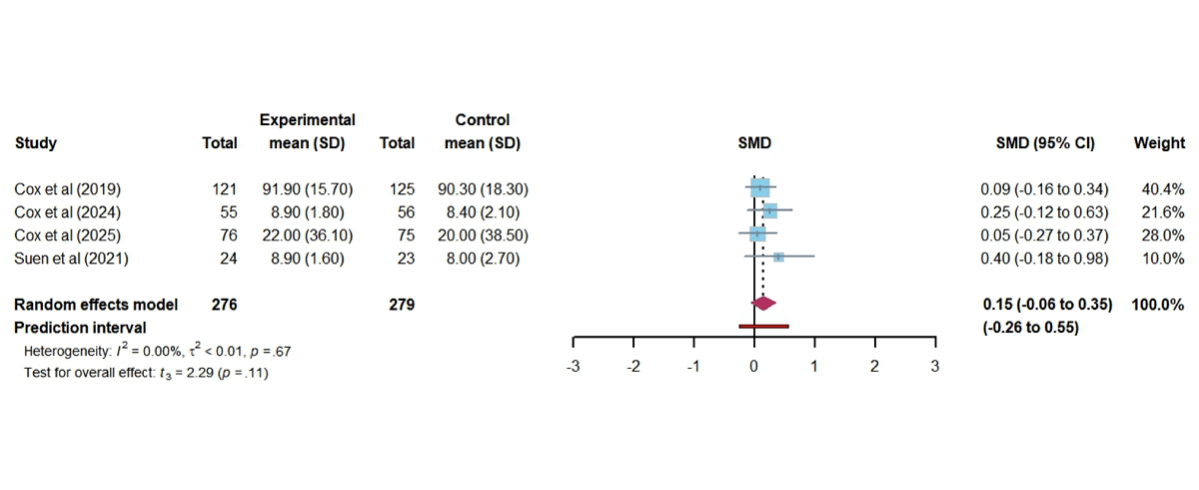
Forest plot of the effectiveness of digital health technology on the quality of communication. SMD: standardized mean difference [41,42,47,52].

In total, 4 studies evaluated the effect of DHIs on the QOC among ICU family members. The pooled analysis showed no statistically significant difference between groups (SMD 0.14; 95% CI –0.03 to 0.31; *P*=.10; Figure 7 [41,42,47,52]). Despite the absence of heterogeneity (*I*^2^=0.00%; τ=0.08; τ^2^=0.01), the actual effect of DHIs on QoL in the future falls within the broader PI range (–0.26 to 0.55), suggesting that future outcomes could vary significantly across different populations and settings. The certainty of this evidence was rated as moderate.

### Subgroup Analysis

We conducted subgroup analyses for the three outcome indicators of anxiety, depression, and PTSD based on potential covariates, including patients’ primary diagnosis, relationship with patients, and type of DHT (Table S1 and Figures S1-S3 in Multimedia Appendix 4). Regarding anxiety, the results of subgroup analyses showed that there was no significant difference (*P*>.05) in the patients’ primary diagnosis (*χ*^2^_2_=2.60; *P*=.27), relationship with patients (*χ*^2^_1_=0.23; *P*=.63), and type of DHT (*χ*^2^_2_=1.43; *P*=.49), suggesting that these factors may not be related to the change of intervention effect. Within the subgroup, family members of medical patients showed a slight improvement trend (SMD –0.14, 95% CI –0.26 to –0.01; *P*=.04). From the perspective of heterogeneity sources, the patients’ primary diagnosis and the type of DHT may be potential sources of heterogeneity. Regarding depression, subgroup analysis showed significant differences between subgroups with different patients’ primary diagnosis (*χ*^2^_2_=29.17; *P*<.01). Among the surgical patients’ family members (3 studies), depression scores in the intervention group were significantly reduced (SMD –0.94, 95% CI –1.64 to –0.24, *t*_2_=–5.80; *P*=.03). In studies focusing on medical patients, no significant effect of the intervention on family members depressive symptoms was observed (SMD –0.04, 95% CI –0.14 to 0.05, *t*_7_=–1.11; *P*=.30). There was no statistical significance (*P*>.05) in the subgroup differences in relationship with patients (*χ*^2^_1_=0.38; *P*=.54) and type of DHT (*χ*^2^_2_=3.11; *P*=.21). Among the subgroups of type of DHT, the effect size of the education-based DHT group (4 studies) had a more minor statistical significance (SMD –0.11, 95% CI –0.21 to –0.01, *t*_3_=–3.47; *P*=.04). Patients’ primary diagnosis and type of DHT may be significant sources of heterogeneity. Regarding PTSD, the differences between subgroups in the patients’ primary diagnosis (*χ*^2^_1_=3.29; *P*=.07), relationship with patients (*χ*^2^_1_=1.30; *P*=.25), and type of DHT (*χ*^2^_2_=2.75; *P*=.25) did not reach statistical significance (*P*>.05). However, looking at the sources of heterogeneity, these factors may be the primary drivers. Due to the limited number of studies in some subgroups, these results should be interpreted with caution.

### Sensitivity Analysis

For anxiety, it had no impact on the statistical significance of the results after removing the study conducted by Xiong et al [16] (SMD –0.18, 95% CI –0.38 to 0.01; *P*=.06; Figure S4 in Multimedia Appendix 4), which decreased heterogeneity to 27.36%. Regarding depression, removing the study by Xiong et al [16] (SMD –0.17, 95% CI –0.38 to 0.05; *P*=.11; Figure S5 in Multimedia Appendix 4) decreased heterogeneity to 36.09%, but it did not alter the statistical significance of the results. For PTSD, sensitivity analyses demonstrated that removing the study by Xiong et al [16] (SMD –0.08, 95% CI –0.21 to 0.05; Figure S6 in Multimedia Appendix 4) resolved heterogeneity, although it did not alter the statistical significance of the results. For QoL, statistical significance was observed only after removing the study by Drop et al [43] (SMD 0.19, 95% CI 0.01 to –0.37; *P*=.04; Figure S7 in Multimedia Appendix 4). Regarding QOC, the effect of DHIs remained stable across studies, indicating robust findings.

### Bias Assessment

Visual inspection of the funnel plots for anxiety, depression, and PTSD showed approximate symmetry (Figures S1-S3 in Multimedia Appendix 5). Egger tests did not provide statistically significant evidence of small-study effects for anxiety (*P*=.24), depression (*P*=.20), and PTSD (*P*=.44; Table S1 in Multimedia Appendix 5). However, we note that small-study effects can arise from various sources beyond publication bias, such as heterogeneity or methodological differences. For other outcomes, there were insufficient numbers of systematic reviews (n<10) to create funnel plots.

## Discussion

### Principal Findings

In total, 17 studies were included, revealing that compared with control groups, DHIs may not confer a significant benefit in reducing anxiety, depression, PTSD, or stress among ICU family members. Moreover, they also did not notably improve self-efficacy, QoL, or communication quality. Given the low certainty of the evidence for the proposed association, the substantial study heterogeneity, and the overall risk of bias in the included studies, the findings should be interpreted with caution. Subgroup analyses indicated that patients’ primary diagnosis may influence the intervention’s effect, with the benefits being more pronounced for families of surgical patients. Patients’ primary diagnosis, relationship with patients, and type of DHT may be a significant source of heterogeneity. Given the PI value, adverse effects of the intervention cannot be ruled out in future studies, and more RCTs are needed to provide robust results.

The observed effect of DHIs on anxiety aligns with previous research concerning improvements in anxiety among caregivers of patients with cancer [23]. The ICU environment is characterized by high stress, restricted access, asymmetric information, and uncertain prognosis, which can significantly increase the psychological burden of family members [54,55]. DHI compensates for the absence of in-person support by using mobile apps, network platforms, and virtual visits [56]. It provides timely updates on diseases, health education, and emotional support for family members, thereby reducing uncertainty and equipping them with knowledge and skills that, in turn, alleviate anxiety symptoms [57]. However, the clinical significance of this finding is tempered by its statistical marginality (*P*=.05), a low certainty of the evidence, and risk of bias in some studies, which significantly reduces the credibility of this potential effect. It is critical to underscore that substantial heterogeneity (*I*^2^=80.19%) yields a wide 95% PI (–1.46 to 0.79), suggesting that the distribution of effects on anxiety is unpredictable. This finding should only be considered a preliminary exploratory conclusion and requires further validation through additional research. No significant effects were observed on depression, PTSD, stress, self-efficacy, QoL, and QOC. It is important to note that the certainty of the evidence for these outcomes is low, with an associated risk of bias, so the reasons for the lack of significant observed effects should be considered from multiple perspectives. From a clinical view, the onset of PTSD is often delayed and complex, typically manifesting months to a year post discharge, making it challenging for short-term interventions and follow-ups to capture long-term effects [14,44]. Additionally, stress, self-efficacy, QOC, and QoL are inherently multidimensional indicators influenced by confounding factors, including economic conditions and social support, beyond psychological state [45,58,59]. From a methodological perspective, existing studies have limitations, including risk of bias, low certainty of the evidence, and imprecision—particularly given that only 2 studies were included for stress and self-efficacy. Current evidence is insufficient to identify the potential benefits or harms of DHIs for these outcomes, and subsequent research is very likely to alter these estimates.

The observed significant heterogeneity across outcomes prompted extensive subgroup analyses. While these analyses pointed to several potential moderators—such as patients’ primary diagnosis and type of DHT for depression—their interpretability is limited. For example, the notable reduction in depression among surgical patients (SMD –0.94, 95% CI –1.64 to –0.24), derived from only 3 studies, may reflect a type Ⅰ error or chance variation rather than an actual differential effect. Similarly, although traditional factors such as family members’ sex and relationship with patients did not reach statistical significance in our analyses, existing literature consistently indicates that female caregivers, especially spouses, often endure greater caregiving stress and more persistent psychological distress [14,60,61]. The overall heterogeneity remained largely unexplained by subgrouping, further underscoring the robustness of these comparisons. Thus, these analyses should not be viewed as confirming specific moderators but rather as highlighting priority variables for future, adequately powered studies.

The limited effectiveness of DHIs may also be attributed to the design of the interventions. Most existing interventions are information-centric, providing unidirectional education rather than incorporating structured psychotherapy, skills training, or facilitating two-way communication [42,62]. Such a format appears insufficient for translating into actual skill enhancement, family interaction, or overall well-being improvement [43]. Furthermore, some studies reported positive subjective feedback from families despite nonsignificant quantitative outcomes, indicating that current measurement tools may lack sensitivity to nuanced experiential gains [40,43,44].

Moving forward, future research should use mixed methods designs to capture subjective experience and identify needs of high-risk subgroups; tailor interventions according to patients’ diagnosis, cultural context, and characteristics of caregiver; develop more interactive and personalized DHIs formats, such as artificial intelligence–based scenario simulations, two-way communication platforms, and longitudinal support programs; extend follow-up duration and use multimodal outcome measures that combine patients and family members reported experiences with clinically relevant indicators; and explore hybrid online-offline interventions to support continuity of care across different phases of the ICU trajectory.

### Strengths and Limitations

This systematic review has several strengths. First, to our knowledge, this is the first meta-analysis to synthesize and appraise the evidence about the effects of DHIs on psychological and related outcomes among family members in the ICU. Second, we strictly followed PRISMA guidelines to strengthen transparency and clarity of reporting. Third, we searched across 9 databases from inception to October 11, 2025, reflecting the most recent advancements in the field of DHI. Moreover, we used the Cochrane RoB 2 and GRADE methodology for quality assessment. Finally, subgroup analyses, sensitivity analyses, and bias assessment were conducted to validate the stability of the results and explore potential sources of heterogeneity.

However, there were several limitations to this review. First, the considerable heterogeneity among studies, as evidenced by the wide PIs for our primary outcomes, indicates uncertainty about the intervention effect. Discrepancies in participants’ characteristics and intervention protocols could lead to inconsistencies and bias in the study findings. Second, the interventions included in the review ranged widely, from simple information websites and SMS text messages to VR- and app-based psychotherapy, and intervention duration varied. Pooling across these fundamentally different modalities may dilute or obscure which components work. Third, while blinding interventionists poses difficulties in DHT care settings, the absence of blinding for study participants and interventionists in most of the included studies introduced risks of bias, resulting in a methodological error. Fourth, the search approach has certain limitations as it exclusively incorporated papers published in English or Chinese, rendering findings from articles in other languages unknown and constraining the global generalizability of the results. Fifth, while no significant small-study effects were detected, the ability of these tests may be constrained by the limited number of included studies. Hence, we cannot conclusively dismiss potential publication bias. Sixth, the majority of the selected trials were conducted in developed countries. This bias may result from the restricted access to technology and the low practicality of technology-based interventions in low-income countries. Seventh, the results regarding the effect of DHIs on psychological outcomes, QoL, and communication quality are inconclusive because of the limited number of studies and the wide PIs. Negligible effects are likely from poor statistical power, not a genuine absence of an intervention effect.

### Implications of Nursing Practice

Given the current limitations and low certainty of the evidence, our findings have limited direct implications for clinical practice but provide valuable insights for future research. ICU nurses, as the medical personnel with the most frequent interactions with patients and their families, are ideally positioned to lead DHIs [23]. They can facilitate online meetings involving physicians, patients, and families to dismantle information barriers, guided by shared decision-making theory. More importantly, nurses can leverage digital platforms to enable personalized communication. This nurse-driven, tailored digital communication strategy not only provides adequate informational support to mitigate their negative emotions but also significantly improves communication quality and overall QoL [63]. The subgroup analyses suggest that DHIs’ effectiveness may not be universal but can be enhanced by tailoring interventions to specific family or patient characteristics. In the future, using technologies such as videoconferencing, virtual simulations, and advanced language models, we can deliver more comprehensive and personalized psychological support interventions led by nurses via the internet and mobile devices. Simultaneously, it is necessary to advocate large-sample, multicenter, high-quality, and reproducible RCTs to provide more robust data to support DHI’s findings on the psychological effects and QoL of ICU family members in China.

### Conclusions

In conclusion, this study provides the first comprehensive synthesis of RCT evidence on DHIs for ICU family members, revealing significant uncertainty in the estimated effects. Despite its methodological rigor and use of PIs, the conclusions of this review are constrained by the intrinsically heterogeneous and low certainty of the existing evidence. Unlike previous reviews, this work integrates more recent studies and focuses explicitly on the family members of ICU patients. Future research should prioritize the development of theory-informed, personalized, and interactive DHIs. Incorporating mixed methods designs and targeting high-risk subgroups would be particularly valuable. Although direct clinical recommendations are limited, DHIs hold potential for enabling more proactive and individualized communication strategies for families of ICU patients.

## Appendix

Multimedia Appendix 1 PRISMA 2020 checklist.

Multimedia Appendix 2 PRISMA-S checklist.

Multimedia Appendix 3 Search strategy.

Multimedia Appendix 4 Subgroup analysis by potential covariates.

Multimedia Appendix 5 Publication bias.

## Abbreviations

CNKI: China Knowledge Resource Integrated Database
DSM-5: Diagnostic and Statistical Manual of Mental Disorders (Fifth Edition)
ICU: intensive care unit
DHI: digital health intervention
DHT: digital health technology
GRADE: Grading of Recommendations, Assessment, Development, and Evaluation
PI: prediction interval
PICS-F: Post-intensive Care Syndrome-Family
PRISMA: Preferred Reporting Items for Systematic Reviews and Meta-analyses
PRISMA-S: Preferred Reporting Items for Systematic Reviews and Meta-analyses literature search extension
PTSD: posttraumatic stress disorder
QOC: quality of communication
QoL: quality of life
RCT: randomized controlled trial
ROB 2: Cochrane’s risk-of-bias tool for randomized trials, version 2
SMD: standardized mean difference
VIP: Weipu Databse
VR: virtual reality

## Data Availability

All data used in this study are available in the article and supplementary material. Further inquiries could be directed to the first author.
