# Supplementary material for: The Effects of Digital Health Interventions for Family Members in Intensive Care Units: Systematic Review and Meta-Analysis of Randomized Controlled Trials
Source: J Med Internet Res. 2026 Feb 25;28:e83294. doi: 10.2196/83294 (PMC12980072; doi:10.2196/83294)
Supplement: Multimedia Appendix 3 [file jmir_v28i1e83294_app3.docx]

**Supplementary materials**

**Multimedia Appendix 3. Search strategy**

| **Database** | **Search Strategies** | **Results** |
| --- | --- | --- |
| CNKI | #1 篇关摘（"智能穿戴设备" + "可穿戴设备" + "应用程序" + "平台" + "移动健康" + "虚拟现实" + "VR" + "增强现实" + "手机" + "网络" + "互联网" + "远程医疗" + "人工智能" + "大数据"） | 6,033,142 |
|  | #2 篇关摘（"重症" + "ICU" + "重症监护室" + "重症监护病房"） | 284,760 |
|  | #3 篇关摘（家属 + 亲属 + "家庭照顾者" + "照护者" + 配偶 + "子女照顾者" + "家庭成员"） | 191,900 |
|  | #4 篇关摘（"随机对照试验" + "随机对照研究" + "RCT" + "随机分配" + "随机分组"） | 1,544,808 |
|  | #5 #1 AND #2 AND #3 AND #4 | 30 |
| Wanfang | #1 主题:("智能穿戴设备" OR "可穿戴设备" OR "应用程序" OR "平台" OR "移动健康" OR "虚拟现实" OR "VR技术" OR "增强现实" OR 手机 OR 网络 OR 互联网 OR "远程医疗" OR "人工智能" OR "大数据") | 7,902,899 |
|  | #2 主题:(重症 OR ICU OR "重症监护室" OR "重症监护病房") | 421,489 |
|  | #3 主题:(家属 OR 亲属 OR "家庭照顾者" OR 照护者 OR 配偶 OR "子女照顾者" OR "家庭成员") | 1,619,258 |
|  | #4 主题:("随机对照试验" OR "随机对照研究" OR RCT OR "随机分配" OR "随机分组") | 387,143 |
|  | #5 #1 AND #2 AND #3 AND #4 | 6 |
| VIP | #1 篇关摘:("智能穿戴设备" OR "可穿戴设备" OR "应用程序" OR "平台" OR "移动健康" OR "虚拟现实" OR "VR技术" OR "增强现实" OR 手机 OR 网络 OR 互联网 OR "远程医疗" OR "人工智能" OR "大数据") | 17,650,949 |
|  | #2 篇关摘:("重症" OR "ICU" OR "重症监护室" OR "重症监护病房") | 323,604 |
|  | #3 篇关摘:("家属" OR "亲属" OR "家庭照顾者" OR "照护者" OR "配偶" OR "子女照顾者" OR "家庭成员") | 209,954 |
|  | #4 篇关摘:("随机对照试验" OR "随机对照研究" OR "RCT" OR "随机分配" OR "随机分组") | 196,781 |
|  | #5 #1 AND #2 AND #3 AND #4 | 26 |
| SinoMed | #1 "智能穿戴设备"[常用字段:智能] OR "可穿戴设备"[常用字段:智能] OR "应用程序"[常用字段:智能] OR "平台"[常用字段:智能] OR "移动健康"[常用字段:智能] OR "虚拟现实"[常用字段:智能] OR "VR技术"[常用字段:智能] OR "增强现实"[常用字段:智能] OR "手机"[常用字段:智能] OR "网络"[常用字段:智能] OR "互联网"[常用字段:智能] OR "远程医疗"[常用字段:智能] OR "人工智能"[常用字段:智能] OR "大数据"[常用字段:智能] | 317,534 |
|  | #2 "重症"[常用字段:智能] OR "ICU"[常用字段:智能] OR "重症监护室"[常用字段:智能] OR "重症监护病房"[常用字段:智能] | 267,300 |
|  | #3 "家属"[常用字段:智能] OR "亲属"[常用字段:智能] OR "照护者"[常用字段:智能] OR "家庭照顾者"[常用字段:智能] OR "配偶"[常用字段:智能] OR "子女照顾者"[常用字段:智能] OR "家庭成员"[常用字段:智能] | 253,853 |
|  | #4 "随机对照试验"[常用字段:智能] OR "随机对照研究"[常用字段:智能] OR "RCT"[常用字段:智能] OR "随机分组"[常用字段:智能] OR "随机分配"[常用字段:智能] | 331,931 |
|  | #5 #1 AND #2 AND #3 AND #4 | 3 |
| Cochrane Library | #1 MeSH descriptor: [Digital Health] explode all trees | 75 |
|  | #2 MeSH descriptor: [Telemedicine] explode all trees | 5,698 |
|  | #3 MeSH descriptor: [Mobile Applications] explode all trees | 2,629 |
|  | #4 MeSH descriptor: [Virtual Reality] explode all trees | 1,586 |
|  | #5 MeSH descriptor: [Artificial Intelligence] explode all trees | 3,736 |
|  | #6 MeSH descriptor: [Telecommunications] explode all trees | 12,672 |
|  | #7 MeSH descriptor: [Remote Consultation] explode all trees | 487 |
|  | #8 MeSH descriptor: [Internet-Based Intervention] explode all trees | 996 |
|  | #9 MeSH descriptor: [computers] explode all trees | 3,136 |
|  | #10 MeSH descriptor: [social media] explode all trees | 662 |
|  | #11 MeSH descriptor: [smartphone] explode all trees | 1,325 |
|  | #12 MeSH descriptor: [electronics] explode all trees | 1,664 |
|  | #13 MeSH descriptor: [blogging] explode all trees | 24 |
|  | #14 MeSH descriptor: [videotape recording] explode all trees | 1,115 |
|  | #15 MeSH descriptor: [telephone] explode all trees | 6,579 |
|  | #16 (digital intervention):ti,ab,kw OR (Computers):ti,ab,kw OR (Smartphone):ti,ab,kw OR (Mobile phone):ti,ab,kw OR (multimedia):ti,ab,kw OR (mobile app):ti,ab,kw OR (mhealth):ti,ab,kw OR (mobile health):ti,ab,kw OR (Internet-Based Intervention):ti,ab,kw OR (web-based):ti,ab,kw OR (on-line communication):ti,ab,kw OR (telecare):ti,ab,kw OR (telehealth):ti,ab,kw OR (tele-monitor):ti,ab,kw OR (ehealth):ti,ab,kw OR (electronic health):ti,ab,kw OR (VR):ti,ab,kw OR (AI):ti,ab,kw OR (Teleconsultation):ti,ab,kw OR (wireless device):ti,ab,kw OR ("Personal Digital Assistant"):ti,ab,kw OR (social media):ti,ab,kw OR (wearables):ti,ab,kw OR (Virtual Medicine):ti,ab,kw OR (Tele Referral):ti,ab,kw OR (Tele Intensive Care):ti,ab,kw | 81,518 |
|  | #17 #1 OR #2 OR #3 OR #4 OR #5 OR #6 OR #7 OR #8 OR #9 OR #10 OR #11 OR #12 OR #13 OR #14 OR #15 OR #16 | 92,716 |
|  | #18 MeSH descriptor: [Critical Care] explode all trees | 3,087 |
|  | #19 MeSH descriptor: [Intensive Care Units] explode all trees | 6,427 |
|  | #20 MeSH descriptor: [Critical Illness] explode all trees | 3,886 |
|  | #21 (Critical Care):ti,ab,kw OR (Intensive Care):ti,ab,kw OR (Surgical Intensive Care):ti,ab,kw OR (Intensive Care Unit):ti,ab,kw OR (ICU):ti,ab,kw OR (Critical Ill):ti,ab,kw OR (critical care unit):ti,ab,kw | 61,930 |
|  | #22 #18 OR #19 OR #20 OR #21 | 62,429 |
|  | #23 MeSH descriptor: [Family] explode all trees | 15,457 |
|  | #24 MeSH descriptor: [Caregivers] explode all trees | 4,193 |
|  | #25 (Family member):ti,ab,kw OR (informal caregiver):ti,ab,kw OR (family caregiver):ti,ab,kw OR (parent):ti,ab,kw OR (spouse):ti,ab,kw OR (caregiver):ti,ab,kw OR (Carers):ti,ab,kw OR (spouse):ti,ab,kw OR (Caretaker):ti,ab,kw OR (Filiation):ti,ab,kw OR (Sibling):ti,ab,kw | 47,545 |
|  | #26 #23 OR #24 OR #25 | 56,802 |
|  | #27 MeSH descriptor: [Randomized Controlled Trial] explode all trees | 34 |
|  | #28 MeSH descriptor: [Controlled Clinical Trials as Topic] explode all trees | 62,560 |
|  | #29 MeSH descriptor: [Clinical Trials] explode all trees | 42 |
|  | #30 (controlled clinical trial):ti,ab,kw OR (controlled trial):ti,ab,kw OR (Clinical trial):ti,ab,kw OR (RCT):ti,ab,kw OR (randomized controlled trial):ti,ab,kw OR (random allocation):ti,ab,kw OR (single blind):ti,ab,kw OR (double blind):ti,ab,kw OR (triple blind):ti,ab,kw | 1,197,427 |
|  | #31 #27 OR #28 OR #29 OR #30 | 1,202,361 |
|  | #32 #17 AND #22 AND #26 AND #31 | 357 |
| PubMed | #1 ("digital health"[MeSH Terms]) OR (telemedicine[MeSH Terms]) OR ("Virtual Reality"[MeSH Terms]) OR ("artificial intelligence"[MeSH Terms]) OR (Telecommunications[MeSH Terms]) OR ("Remote Consultation"[MeSH Terms]) OR ("Mobile Applications"[MeSH Terms]) OR ("Internet-Based Intervention"[MeSH Terms]) OR (computers[MeSH Terms]) OR ("social media"[MeSH Terms]) OR (smartphone[MeSH Terms]) OR (electronics[MeSH Terms]) OR (blogging[MeSH Terms]) OR ("videotape recording"[MeSH Terms]) OR (technology[MeSH Terms]) OR (telephone[MeSH Terms]) | 981,169 |
|  | #2 ("digital health"[Title/Abstract]) OR (telemedicine[Title/Abstract]) OR ("mobile health"[Title/Abstract]) OR ("internet based intervention"[Title/Abstract]) OR ("mobile application*"[Title/Abstract]) OR ("online intervention"[Title/Abstract]) OR (telephone[Title/Abstract]) OR (phon*[Title/Abstract]) OR (Smartphone[Title/Abstract]) OR ("Mobile phone"[Title/Abstract]) OR (telecare[Title/Abstract]) OR (telehealth*[Title/Abstract]) OR (tele-monitor*[Title/Abstract]) OR ("videotape recording"[Title/Abstract]) OR (video*[Title/Abstract]) OR (blog*[Title/Abstract]) OR (ehealth[Title/Abstract]) OR ("electronic health*"[Title/Abstract]) OR (mhealth[Title/Abstract]) OR (web-based[Title/Abstract]) OR ("online communication"[Title/Abstract]) OR (Comput*[Title/Abstract]) OR (multimedia[Title/Abstract]) OR ("social media"[Title/Abstract]) OR (wearables[Title/Abstract]) OR ("Virtual Medicine"[Title/Abstract]) OR ("Tele Referral"[Title/Abstract]) OR ("technolog*"[Title/Abstract]) OR (VR[Title/Abstract]) OR (AI[Title/Abstract]) OR (Teleconsultation[Title/Abstract]) OR ("wireless device"[Title/Abstract]) OR ("Personal Digital Assistant*"[Title/Abstract]) OR ("digital intervention*"[Title/Abstract]) OR (remote consultation[Title/Abstract]) | 2,590,654 |
|  | #3 #1 OR #2 | 3,216,428 |
|  | #4 ("Critical Care"[MeSH Terms]) OR ("Intensive Care Units"[MeSH Terms]) OR ("Critical Illness"[MeSH Terms]) | 195,173 |
|  | #5 ("Critical Care"[Title/Abstract]) OR ("Intensive Care"[Title/Abstract]) OR ("Surgical Intensive Care"[Title/Abstract]) OR ("Intensive Care Unit"[Title/Abstract]) OR (ICU[Title/Abstract]) OR ("Critical Ill*"[Title/Abstract]) OR ("critical care unit"[Title/Abstract]) | 304,791 |
|  | #6 #4 OR #5 | 368,126 |
|  | #7 (Family[MeSH Terms]) OR (Caregivers[MeSH Terms]) | 437,846 |
|  | #8 ("Family member*"[Title/Abstract]) OR ("informal caregiver*"[Title/Abstract]) OR ("family caregiver*"[Title/Abstract]) OR (parent*[Title/Abstract]) OR (spouse[Title/Abstract]) OR (caregiver*[Title/Abstract]) OR (Carers[Title/Abstract]) OR (Caretaker*[Title/Abstract]) OR (Relative*[Title/Abstract]) OR (Filiation[Title/Abstract]) OR (Sibling*[Title/Abstract]) | 2,600,589 |
|  | #9 #7 OR #8 | 2,809,502 |
|  | #10 ("Clinical Trial"[Publication Type]) OR ("Controlled Clinical Trial"[Publication Type]) OR ("Randomized Controlled Trial"[Publication Type]) | 1,034,594 |
|  | #11 ("Randomized Controlled Trials as Topic"[MeSH Terms]) OR ("Controlled Clinical Trials as Topic"[MeSH Terms]) OR ("Random Allocation"[MeSH Terms]) OR ("Single-Blind Method"[MeSH Terms]) OR ("Double-Blind Method"[MeSH Terms]) | 504,151 |
|  | #12 ("randomized controlled trial*"[Title/Abstract]) OR ("controlled trial"[Title/Abstract]) OR (RCT[Title/Abstract]) OR (random*[Title/Abstract]) OR ("trial"[Title/Abstract]) OR ("clinical trial*"[Title/Abstract]) OR ("single blind*"[Title/Abstract]) OR ("double blind*"[Title/Abstract]) OR ("triple blind*"[Title/Abstract]) | 2,367,510 |
|  | #13 #10 OR #11 OR #12 | 2,850,305 |
|  | #14 #3 AND #6 AND #9 AND #13 | 799 |
| Embase | #1 'digital health'/exp | 6,765 |
|  | #2 'telemedicine'/exp | 91,690 |
|  | #3 'virtual reality'/exp | 38,051 |
|  | #4 'artificial intelligence'/exp | 163,182 |
|  | #5 'telecommunication'/exp | 161,649 |
|  | #6 'teleconsultation'/exp | 19,106 |
|  | #7 'mobile application'/exp | 37,599 |
|  | #8 'web-Based Intervention'/exp | 5,706 |
|  | #9 'Computer'/exp | 199,850 |
|  | #10 'social media'/exp | 72,115 |
|  | #11 'Smartphone'/exp | 41,293 |
|  | #12 'Electronics'/exp | 95,433 |
|  | #13 'Blogging'/exp | 1,232 |
|  | #14 'video recording'/exp | 148,675 |
|  | #15 'telephone'/exp | 54,468 |
|  | #16 'digital health':ti,ab OR 'telemedicine':ti,ab OR 'telecare':ti,ab OR 'telehealth':ti,ab OR 'tele-monitor':ti,ab OR 'ehealth':ti,ab OR 'electronic health':ti,ab OR 'mhealth':ti,ab OR 'mobile health':ti,ab OR 'internet-based':ti,ab OR 'web-based':ti,ab OR 'online intervention':ti,ab OR 'computer':ti,ab OR 'telephone':ti,ab OR 'smartphone':ti,ab OR 'mobile phone':ti,ab OR 'multimedia':ti,ab OR 'mobile application':ti,ab OR 'social media':ti,ab OR 'wearables':ti,ab OR 'virtual medicine':ti,ab OR 'teleconsultation':ti,ab OR 'wireless device':ti,ab OR 'personal digital assistant':ti,ab OR 'digital intervention':ti,ab OR 'video recording':ti,ab OR 'video':ti,ab OR 'blog':ti,ab OR 'VR':ti,ab OR 'AI':ti,ab OR 'remote consultation':ti,ab | 1,104,179 |
|  | #17 #1 OR #2 OR #3 OR #4 OR #5 OR #6 OR #7 OR #8 OR #9 OR #10 OR #11 OR #12 OR #13 OR #14 OR #15 OR #16 | 1,556,662 |
|  | #18 'critical care'/exp | 1,045,682 |
|  | #19 'intensive care unit'/exp | 386,794 |
|  | #20 'critical illness'/exp | 41,514 |
|  | #21 'critical care':ti,ab OR 'surgical intensive care':ti,ab OR 'intensive care unit':ti,ab OR 'intensive care':ti,ab OR 'surgical intensive care':ti,ab OR 'icu':ti,ab OR 'critical ill*':ti,ab OR 'critical care unit':ti,ab | 493,661 |
|  | #22 #18 OR #19 OR #20 OR #21 | 1,406,467 |
|  | #23 'family'/exp | 693,058 |
|  | #24 'caregiver'/exp | 152,786 |
|  | #25 'family member*':ti,ab OR 'informal caregiver*':ti,ab OR 'family caregiver*':ti,ab OR 'parent*':ti,ab OR caregiver*:ti,ab OR 'carers':ti,ab OR 'spouse':ti,ab OR 'caretaker':ti,ab OR 'filiation':ti,ab OR 'sibling*':ti,ab | 1,101,580 |
|  | #26 #23 OR #24 OR #25 | 1,516,534 |
|  | #27 'clinical trial'/de OR 'controlled clinical trial'/de OR 'randomized controlled trial' OR 'controlled clinical trial (topic)'/exp OR 'random allocation'/exp OR 'double-blind method'/exp OR 'single blind procedure'/exp | 2,701,942 |
|  | #28 'randomized controlled trial*':ti,ab OR 'rct':ti,ab OR 'clinical trial*':ti,ab OR 'single blind*':ti,ab OR 'double blind*':ti,ab OR 'triple blind*':ti,ab | 1,501,554 |
|  | #29 #27 OR #28 | 3,257,303 |
|  | #30 #17 AND #22 AND #26 AND #29 | 876 |
| CINAHL | #1 SU('digital health' OR 'telemedicine' OR 'telecare' OR 'telehealth' OR 'tele-monitor' OR 'ehealth' OR 'electronic health' OR 'mhealth' OR 'mobile health' OR 'internet-based' OR 'web-based' OR 'online intervention' OR 'computer' OR 'telephone' OR 'smartphone' OR 'mobile phone' OR 'multimedia' OR 'mobile application' OR 'social media' OR 'wearables' OR 'virtual medicine' OR 'teleconsultation' OR 'wireless device' OR 'personal digital assistant' OR 'digital intervention' OR 'video recording' OR 'video' OR 'blog' OR 'VR' OR 'AI' OR 'remote consultation' OR 'telecommunication' OR 'artificial intelligence' OR 'virtual reality') | 295,355 |
|  | #2 SU('critical care' OR 'surgical intensive care' OR 'intensive care unit' OR 'intensive care' OR 'surgical intensive care' OR 'icu' OR 'critical ill*' OR 'critical care unit' OR 'critical illness') | 129,802 |
|  | #3 SU('family member*' OR 'informal caregiver*' OR 'family caregiver*' OR 'parent*' OR caregiver* OR 'carers' OR 'spouse' OR 'caretaker' OR 'filiation' OR 'sibling*') | 206,234 |
|  | #4 SU('controlled clinical trial' OR 'random allocation' OR 'randomized controlled trial*' OR 'rct' OR 'clinical trial*' OR 'single blind*' OR 'double blind*' OR 'triple blind*') | 363,649 |
|  | #5 #1 OR #2 OR #3 OR #4 | 17 |
| Web of Science | #1 TS=("digital health" OR telemedicine OR telecare OR telehealth* OR tele-monitor* OR ehealth OR "electronic health*" OR mhealth OR "mobile health" OR "Internet-Based Intervention" OR web-based OR "online intervention" OR Computer OR Smartphone OR telephone OR "Mobile phone" OR multimedia OR "mobile applications" OR "social media" OR wearables OR "Virtual Medicine" OR "Tele Referral" OR electronics OR "Virtual Reality" OR VR OR AI OR "artificial intelligence" OR Telecommunications OR "Remote Consultation" OR Teleconsultation OR technology OR "wireless device" OR "Personal Digital Assistant*" OR "digital intervention*" OR "videotape recording" OR video OR blogging OR blog) | 2,443,129 |
|  | #2 TS=("Critical Care" OR "Intensive Care" OR "Surgical Intensive Care" OR "Intensive Care Units" OR ICU OR "Critical Ill*" OR "critical care unit" OR "Critical Illness") | 224,968 |
|  | #3 TS=(Family OR Caregivers OR "Family member*" OR "informal caregiver*" OR "family caregiver*" OR parent* OR spouse OR caregiver* OR Carers OR spouse OR Caretaker* OR Relative* OR Filiation OR Sibling*) | 3,307,055 |
|  | #4 TS=("randomized controlled trial*" OR "clinical trial" OR RCT OR random* OR trial OR "single blind*" OR "double blind*" OR "triple blind*") | 2,580,111 |
|  | #5 #1 AND #2 AND #3 AND #4 | 522 |
