# Supplementary material for: The Effects of Digital Health Interventions for Family Members in Intensive Care Units: Systematic Review and Meta-Analysis of Randomized Controlled Trials
Source: J Med Internet Res. 2026 Feb 25;28:e83294. doi: 10.2196/83294 (PMC12980072; doi:10.2196/83294)
Supplement: Multimedia Appendix 4 [file jmir_v28i1e83294_app4.docx]

**Supplementary materials**

**Multimedia Appendix 5.**

**Table1. Subgroup analysis by potential covariates**

| **Outcomes** | **Subgroup** | **Studies** | **I^2^(%)** | **SMD** | **95% CI** | ***χ^2^*-value** | ***df*** | ***P*-value** |
| --- | --- | --- | --- | --- | --- | --- | --- | --- |
| Anxiety | | 12 | 80.19 | -0.34 | [-0.68, 0.00]  ] |  |  |  |
|  | **Patients primary diagnosis** |  |  |  |  | 2.60 | 2 | 0.27 |
|  | Medical | 7 | 0 | -0.14 | **[-0.26, -0.01]** |  |  | **0.04** |
|  | Surgical | 4 | 91.98 | -0.78 | [-2.08, 0.52] |  |  | 0.15 |
|  | Not reported | 1 | - | -0.06 | [-0.52, 0.40] |  |  | - |
|  | **Relationship with patient** |  |  |  |  | 0.23 | 1 | 0.63 |
|  | Children | 6 | 87.96 | -0.40 | [-1.10, 0.30] |  |  | 0.20 |
|  | Spouse | 6 | 53.56 | -0.25 | [-0.67, 0.18] |  |  | 0.19 |
|  | **Type of DHT** |  |  |  |  | 1.43 | 2 | 0.48 |
|  | Education-based DHT | 3 | 0 | -0.17 | [-0.49, 0.15] |  |  | 0.15 |
|  | Communication-based DHT | 6 | 56.81 | -0.19 | [-0.63, 0.25] |  |  | 0.32 |
|  | Mental health-related DHT | 3 | 91.67 | -0.77 | [-2.90, 1.36] |  |  | 0.26 |
| Depression | | 12 | 67.80 | -0.26 | [-0.52, 0.01] |  |  |  |
|  | **Patients primary diagnosis** |  |  |  |  | **29.17** | **2** | **＜0.01** |
|  | Medical | 8 | 0 | -0.04 | [-0.14, 0.05] |  |  | 0.30 |
|  | Surgical | 3 | 8.59 | -0.94 | **[-1.64, -0.24]** |  |  | **0.03** |
|  | Not reported | 1 | - | -0.22 | [-0.68, 0.24] |  |  | - |
|  | **Relationship with patient** |  |  |  |  | 0.38 | 1 | 0.54 |
|  | Children | 5 | 73.54 | -0.34 | [-0.96, 0.17] |  |  | 0.14 |
|  | Spouse | 7 | 60.75 | -0.19 | [-0.60, 0.22] |  |  | 0.30 |
|  | **Type of DHT** |  |  |  |  | 3.11 | 2 | 0.21 |
|  | Education-based DHT | 4 | 0 | -0.11 | **[-0.21, -0.01]** |  |  | **0.04** |
|  | Communication-based DHT | 5 | 65.23 | -0.16 | [-0.78, 0.47] |  |  | 0.52 |
|  | Mental health-related DHT | 3 | 76.06 | -0.62 | [-1.85, 0.61] |  |  | 0.16 |
| PTSD | | 11 | 69.98 | -0.21 | [-0.49, 0.06] |  |  |  |
|  | **Patients primary diagnosis** |  |  |  |  | 3.29 | 1 | 0.07 |
|  | Medical | 9 | 0 | -0.06 | [-0.18, 0.07] |  |  | 0.33 |
|  | Surgical | 2 | 84.25 | -0.86 | [-6.41, 4.70] |  |  | 0.30 |
|  | **Relationship with patient** |  |  |  |  | 1.30  .3 | 1 | 0.25 |
|  | Children | 4 | 86.14 | -0.40 | [-1.36, 0.55] |  |  | 0.27 |
|  | Spouse | 7 | 0 | -0.05 | [-0.23, 0.12] |  |  | 0.50 |
|  | **Type of DHT** |  |  |  |  | 2.75 | 2 | 0.25 |
|  | Education-based DHT | 3 | 0 | -0.04 | [-0.12, 0.04] |  |  | 0.15 |
|  | Communication-based DHT | 4 | 26.16 | -0.07 | [-0.44, 0.29] |  |  | 0.57 |
|  | Mental health-related DHT | 4 | 81.67 | -0.54 | [-1.49, 0.42] |  |  | 0.17 |

**2.Subgroup Analyses of Anxiety**

**
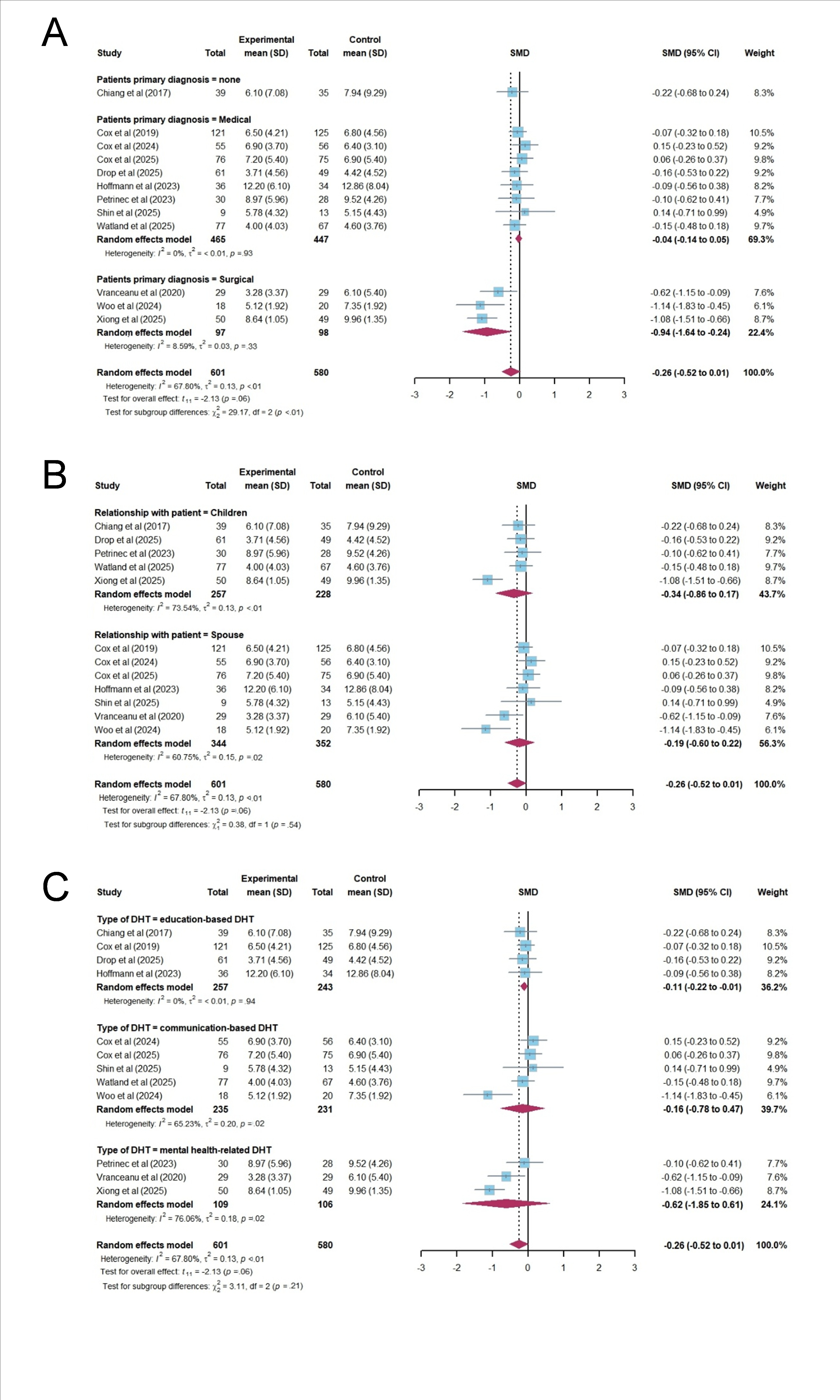
**

Figure 1 Subgroup Analysis of Anxiety According to A) Patients primary diagnosis; B) Relationship with patient; C) Type of DHT.

**3.Subgroup Analysis of Depression**

**
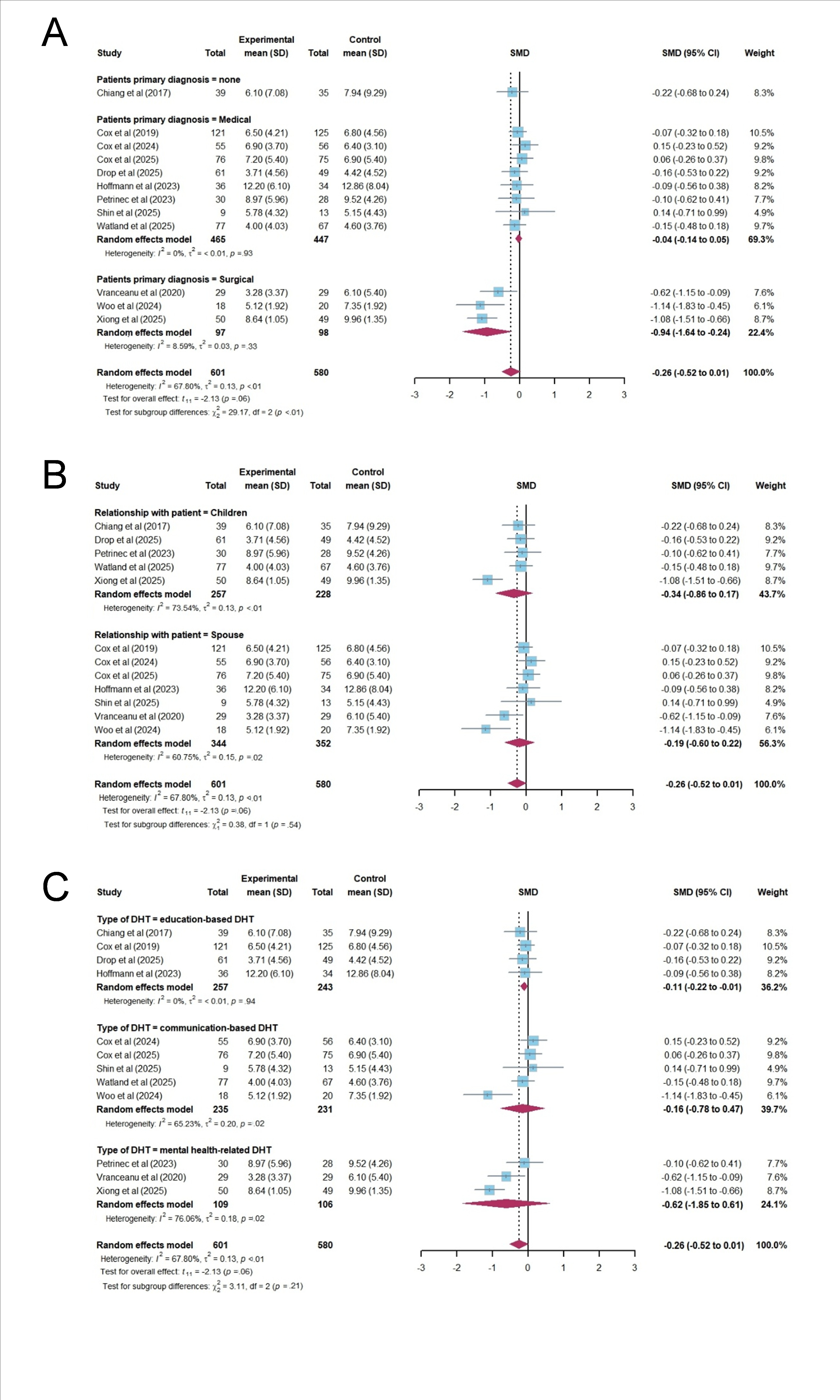
**

Figure 2 Subgroup Analysis of Depression According to A) Patients primary diagnosis; B) Relationship with patient; C) Type of DHT.

**4.Subgroup Analysis of PTSD**

**
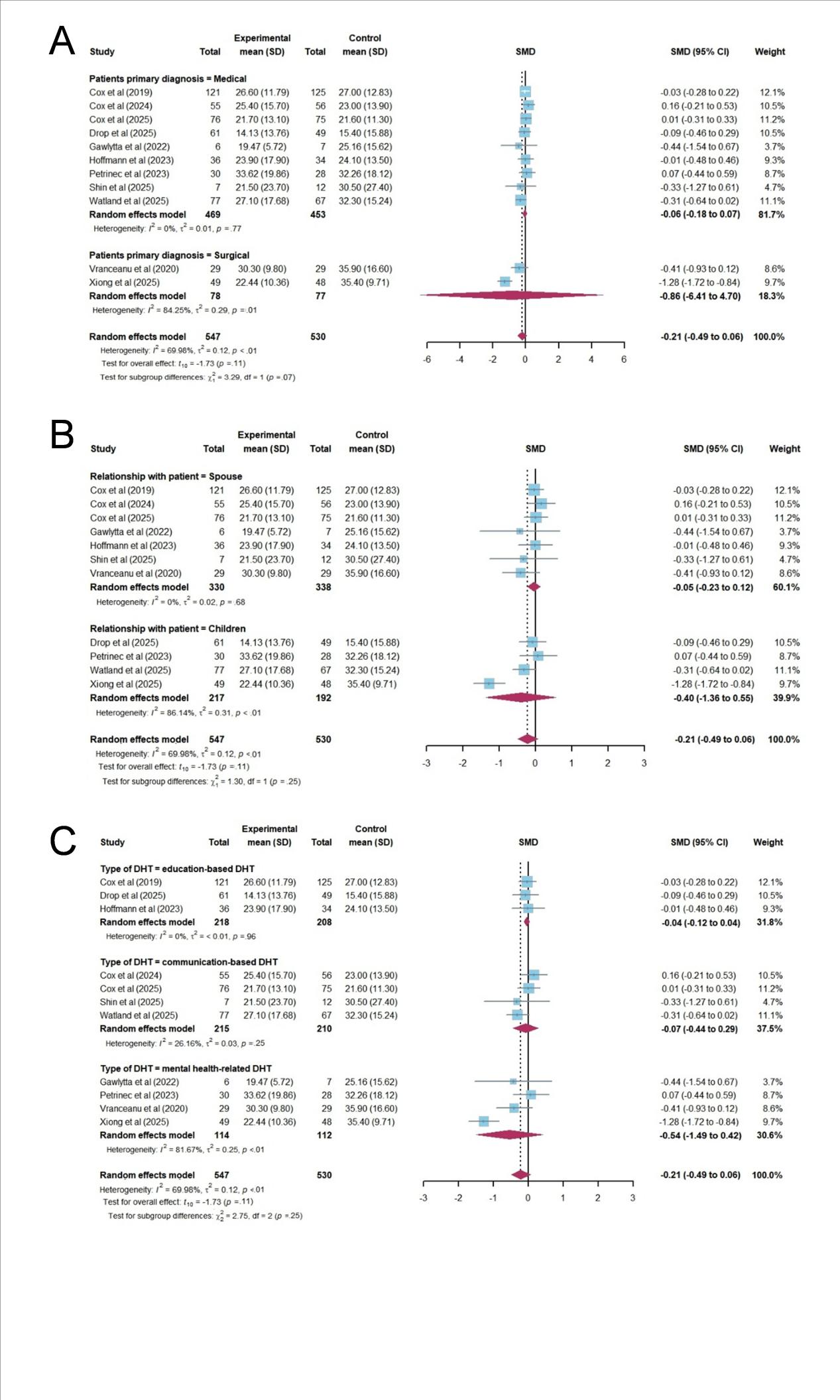
**

Figure 3 Subgroup Analysis of PTSD According to A) Patients primary diagnosis; B) Relationship with patient; C) Type of DHT.

**5.Sensitivity Analysis**

**
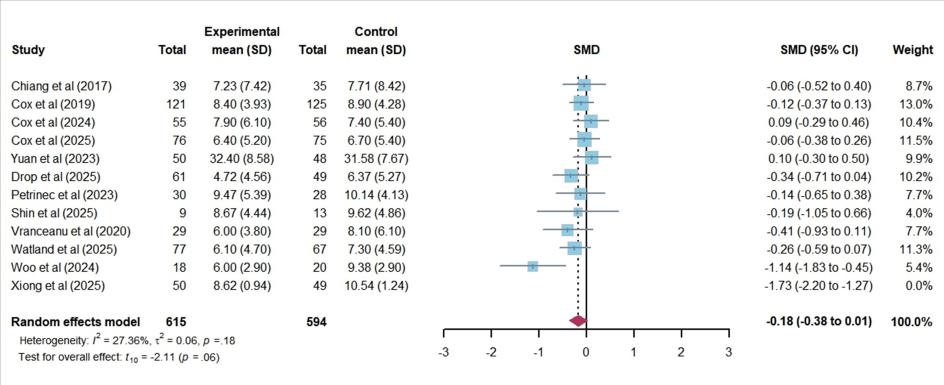
**

Figure 4 Sensitivity Analysis of Anxiety Meta-Analysis: Forest Plot After Excluding the Study by Xiong et al (2025)


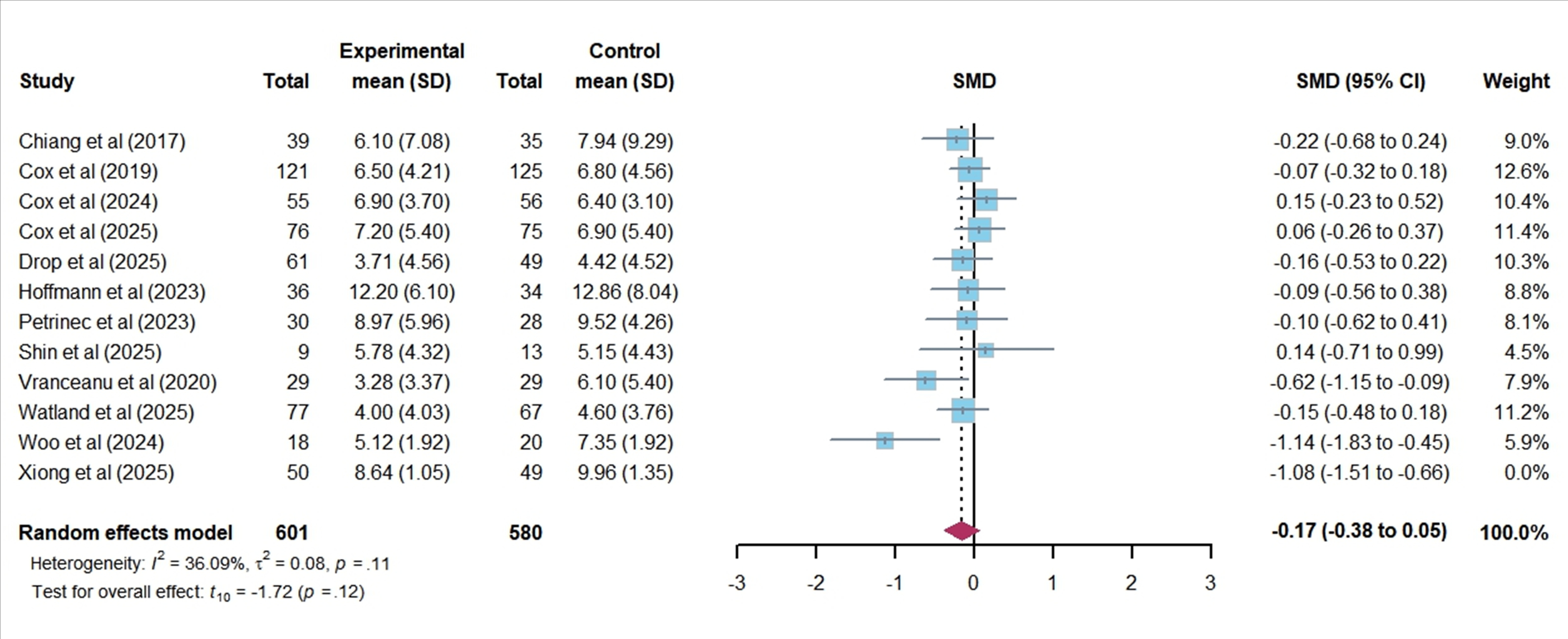


Figure 5 Sensitivity Analysis of Depression Meta-Analysis: Forest Plot After Excluding the Study by Xiong et al (2025)


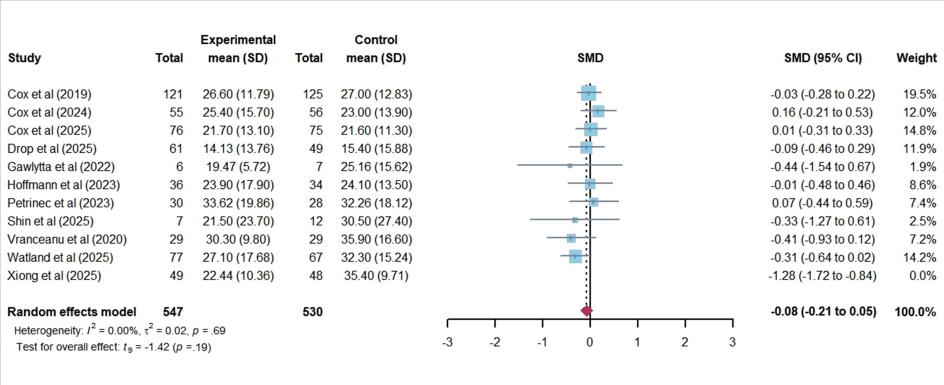


Figure 6 Sensitivity Analysis of PTSD Meta-Analysis: Forest Plot After Excluding the Study by Xiong et al (2025)


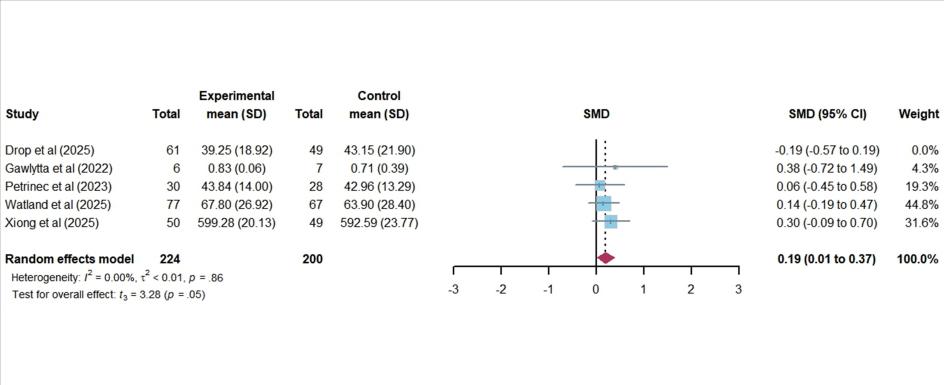


Figure 7 Sensitivity Analysis of QoL Meta-Analysis: Forest Plot After Excluding the Study by Drop et al (2025)
