# Supplementary material for: The Effects of Digital Health Interventions for Family Members in Intensive Care Units: Systematic Review and Meta-Analysis of Randomized Controlled Trials
Source: J Med Internet Res. 2026 Feb 25;28:e83294. doi: 10.2196/83294 (PMC12980072; doi:10.2196/83294)
Supplement: Multimedia Appendix 5 [file jmir_v28i1e83294_app5.docx]

**Supplementary materials**

**Multimedia Appendix 6.**
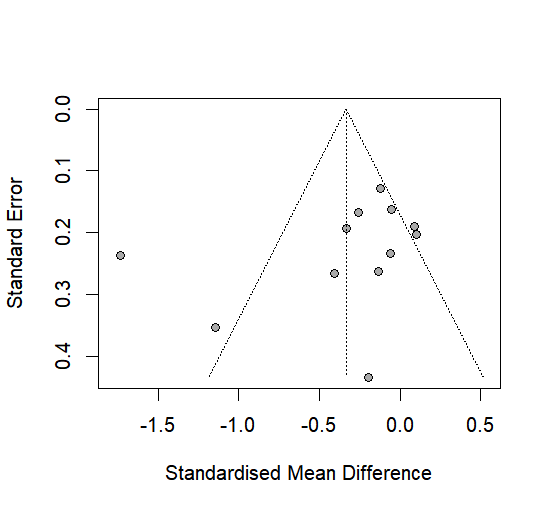


**Figure 1 Funnel plot for anxiety.**


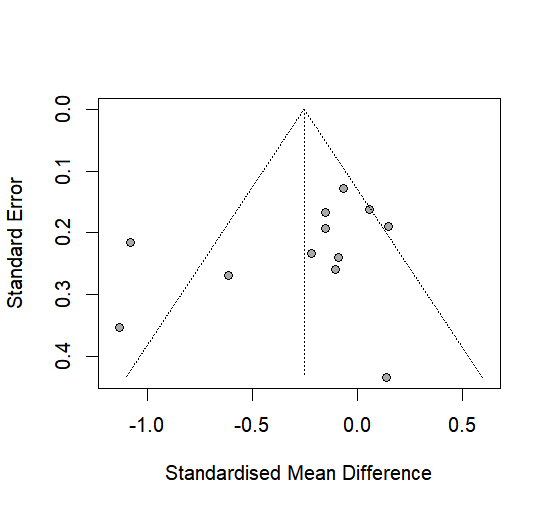


**Figure 2 Funnel plot for depression.**


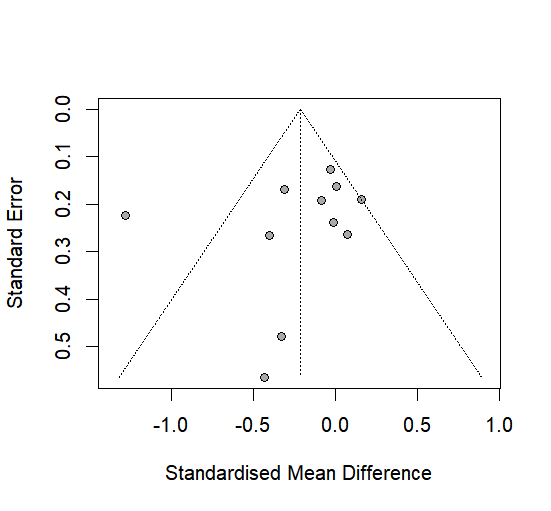


**Figure 3 Funnel plot for PTSD.**

**Table 1 Egger’s test**

|  | Bias estimate | p-value |
| --- | --- | --- |
| anxiety | -2.70 (SE = 2.14) | 0.24 |
| depression | -2.25 (SE = 1.65) | 0.20 |
| PTSD | -1.31 (SE = 1.62) | 0.44 |
